# Validity of patient-derived xenograft mouse models for lung cancer based on exome sequencing data

**DOI:** 10.5808/GI.2020.18.1.e3

**Published:** 2020-03-31

**Authors:** Jaewon Kim, Hwanseok Rhee, Jhingook Kim, Sanghyuk Lee

**Affiliations:** 1 Department of Bio-information Science, Ewha Womans University, Seoul 03760, Korea; 2 Bioinformatics Team, DNA Link, Seoul 03759, Korea; 3 Samsung Biomedical Research Institute, Samsung Medical Center, Sungkyunkwan University School of Medicine, Seoul 06351, Korea; 4 Ewha Research Center for Systems Biology (ERCSB) and Department of Life Science, Ewha Womans University, Seoul 03760, Korea

**Keywords:** copy number alteration, lung neoplasms, mutation, patient-derived xenograft, whole exome sequencing

## Abstract

Patient-derived xenograft (PDX) mouse models are frequently used to test the drug efficacy in diverse types of cancer. They are known to recapitulate the patient characteristics faithfully, but a systematic survey with a large number of cases is yet missing in lung cancer. Here we report the comparison of genomic characters between mouse and patient tumor tissues in lung cancer based on exome sequencing data. We established PDX mouse models for 132 lung cancer patients and performed whole exome sequencing for trio samples of tumor-normal-xenograft tissues. Then we computed the somatic mutations and copy number variations, which were used to compare the PDX and patient tumor tissues. Genomic and histological conclusions for validity of PDX models agreed in most cases, but we observed eight (~7%) discordant cases. We further examined the changes in mutations and copy number alterations in PDX model production and passage processes, which highlighted the clonal evolution in PDX mouse models. Our study shows that the genomic characterization plays complementary roles to the histological examination in cancer studies utilizing PDX mouse models.

## Introduction

Lung cancer incidence and mortality rates are the highest worldwide, accounting for 11.6% of the total cases and 18.4% of the total cancer deaths in 2018 [1]. Traditional treatment for lung cancer has been surgery and radiochemotherapy, but targeted therapies are increasingly adopted for patients who have the druggable aberrations such as epidermal growth factor receptor (*EGFR*) mutations or gene fusions involving *ALK, ROS1*, and *NTRK* genes [2-5]. Targeted therapies usually show fast response with minimal side effects, but tumor recurs within a few months in many cases, thus necessitating additional therapies.

The main reasons for resistant and recurrent tumors are intrinsic heterogeneity and tumor cell evolution. Tumors may consist of multiple clones where targeted therapies kill only subsets of clones leaving residual clones, whose proliferation leads to resistance or recurrence eventually. Alternatively, tumor cells may undergo evolution after treatment to acquire *de novo* mutations overcoming the treatment effect of cancer drugs. Understanding molecular mechanisms of resistance development is essential to identify follow-up treatment options in targeted therapy.

Preclinical models are extremely useful in the course of drug development, especially to test the drug efficacy in cost-effective ways. Animal models and organoids derived from patient tumors are two representative systems frequently adopted in cancer drug development. Patient-derived xenograft (PDX) mouse models where the patient’s tumor tissue is transplanted into immunodeficient mice have demonstrated their usefulness to recapitulate patient’s response to cancer agents in various types of cancers including breast, brain, colon, and lung tumors [6-10]. The banks of these ‘Avatar’ mice are valuable resources for preclinical tests. However, the fidelity of PDX mouse models to substitute patient’s tumor tissues has not been thoroughly studied in lung cancer models. Here we compare the genomic characteristics of PDX mouse, patient tumor, and patient normal tissues based on exome sequencing data to test the validity of PDX mouse models in lung cancer.

## Methods

### Producing whole exome sequencing data

We acquired the tumor and matched normal tissues from 132 lung cancer patients at the Samsung Medical Center in Seoul. This study was approved by Samsung Medical Center institutional review board (IRB 2018-03-110), and informed consent was obtained from each patient. Tumor tissues were transplanted into the NSG mouse, NOD.Cg-Prkdc^scid^Il2rg^tm1Wjl^/SzJ (Stock No. 005557) [11], purchased from the Jackson Laboratory to establish the PDX mouse models. All tumor tissues of the patient and PDX were prepared for formalin-fixed paraffin-embedded, and a pathologist examined histopathology of tissue through hematoxylin and eosin staining. Whole exome sequencing (WES) was performed using the Illumina TruSeq Exome kit and HiSeq 2500 sequencing platform (Illumina, San Diego, CA, USA). The WES data for patient tumor, patient normal, and PDX tumor tissues were deposited at the Korean Bioinformation Center (KOBIC) (ID 10050154).

### Data processing and variant analysis

Preprocessing steps consist of adapter trimming, quality control, and filtering mouse reads. First, adapter sequences were removed and sequence reads whose quality score < 33 in more than 50% of bases were discarded using fastx-toolkit (ver. 0.0.14). After the trimming process, we removed the single reads and kept the paired-end reads only using cmpfastq perl program (http://compbio.brc.iop.kcl.ac.uk/software/cmpfastq.php). For WES data from PDX mouse tissues, we applied an additional step to filter out mouse-originated reads using Xenome software (ver. 1.0.1) [12] with the reference genomes of human (UCSC hg19 in https://genome.ucsc.edu/) and mouse (UCSC mm10). We kept the human-specific reads only for subsequent analyses.

Resulting reads were mapped to the human reference genome (UCSC hg19) using Burrows-Wheeler Alignment (BWA)-MEM alignment tool [13] with default options. After mapping, reads were sorted by samtools version 1.8 [14]. We performed Genome Analysis Toolkit (GATK, v4.0.7.0) [15] AddOrReplaceReadGroups command for adding read group information, MarkDuplicates command for removing duplicated reads, BaseRecalibrator and ApplyBQSR commands for correcting realignment. Data processing summary statistics are given in Supplementary Table 1. Then, somatic single nucleotide variations (SNVs) and insertions/deletions were called using the GATK4-Mutect2 [16] pipeline. Filter-based annotation in ANNOVAR [17] was used for variant annotations. In addition, we calculated the copy number alterations using EXCAVATOR2 (v1.1.2) [18]. All statistical analysis and visualizations were performed using R version 3.6.1

## Results

### Clinical and histopathological features of samples

We analyzed 132 lung cancer patients whose tumor and matched normal tissues were available and the PDX tumor samples were successfully harvested. The pathophysiological information of patients is summarized in Table 1. Briefly, we had 54 adenocarcinoma cases (41%), 48 squamous cell carcinoma cases (36%), four large cell carcinoma cases (3%), and 26 unclassifiable adenocarcinoma cases (20%). Our patient cohort was enriched with male (66%), smokers (64%), early stages (50%), non-recurrent (61%), and primary (62%) patients. In accordance with the previous reports [19], the success rate of establishing PDX mouse models was higher in squamous cell carcinoma than in adenocarcinoma. Histopathological examination of patient tumor and PDX tumor tissues concluded that tumors were consistent between patient and PDX mouse in 97 cases (73.5%). The discrepancy in histology maybe presumably ascribed to the lymphomagenesis that had been reported to occur frequently in NSG or NOG mice transplanted with Epstein-Barr virus infected tumor tissues [20,21]. This concordance and discrepancy were further investigated by comparing the mutation and copy number profiles between patient and PDX mouse tumors.

### Statistics in mapping and variant calling procedure

**Fig. 1.**
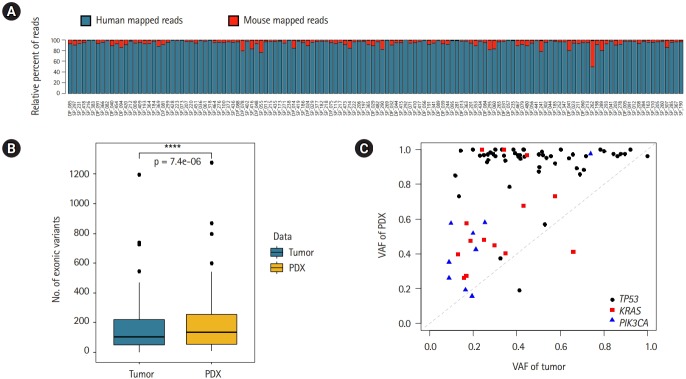
Mapping and somatic mutations of patient-derived xenograft (PDX) mouse models. (A) The percentage of reads mapped to human and mouse respectively in PDX. (B) Box plot comparison of the number of exonic mutations called in patient and PDX tumors. (C) Variant allele frequencies (VAF) of major mutations identified in both patient and PDX tumors.

We performed WES on tumor, normal, and PDX samples of 132 lung cancer patients, with a mean coverage of 30×. In order to compare the somatic mutation profiles of patient and PDX mouse tumors, it is essential to test the reliability of mutation calls in the PDX mouse tumors because human stromal cells are replaced with the mouse stromal cells during engraftment. Thus, we checked the portion of mouse-originated reads from the WES data of PDX tumors using the Xenome software to separate the human and mouse reads specifically. A summary of the Xenome alignment results is provided in Supplementary Table 2. The median portion of human reads was 95.1% and that of mouse reads was below 5% except a few cases (Fig. 1A). This implied that the PDX mouse tumors contained a sufficient amount of human cells. Thus we used the well-known MuTect2 algorithm after BWA-MEM mapping to call somatic mutations in PDX mouse tumors.

The mutation rates were significantly higher in PDX mouse tumors than in patient tumors (p = 7.36e-06). The median values of exonic mutations in PDX and patient tumors were 136 and 102, respectively (Fig. 1B). Since we removed the mouse-originated reads before calling somatic mutations, the difference can be attributed to clonal selection and evolutionary processes in establishing PDX mouse tumors. For example, human stromal cells would be replaced with the mouse stromal cells during engraftment of the patient's tumor tissue into immunodeficient mice [22]. As a result, tumor cells are enriched in the PDX mouse tumors, which leads to more somatic mutations in variant calling. To test this hypothesis, we examined the variant allele frequencies (VAFs) of major cancer genes (*TP53, KRAS*, and *PIK3CA*) identified in both patient and PDX tumors (Fig. 1C). All three genes showed that the VAFs of PDX mouse tumors are larger than those of patient tumors, which supports our hypothesis of clonal enrichment in PDX mouse tumors. However, the extent of clonal selection pressure varied for different genes. Interestingly, the VAFs of *TP53* gene were close to 100% in PDX mouse tumors. The biological meaning of this observation warrants further studies.

### Comparison of somatic mutations between patient and PDX mouse tumors

**Fig. 2.**
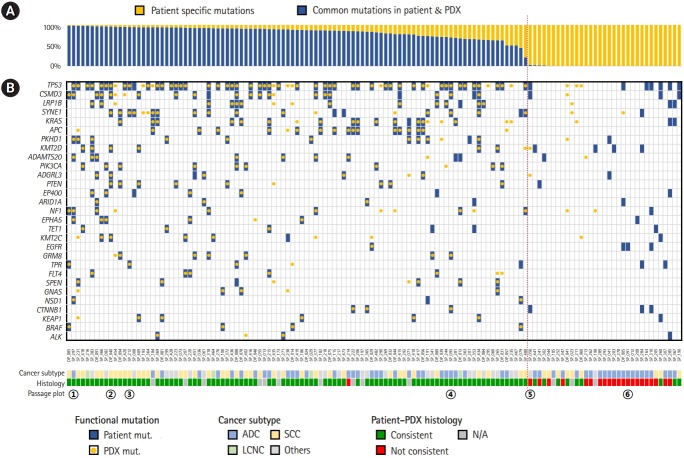
Landscape of somatic mutations in lung cancer patients and patient-derived xenograft (PDX) mouse models. (A) Relative ratio of exonic mutations identified in patients. Somatic mutations in patients were divided into patient-specific ones and common ones in PDX tumors. (B) Mutational landscape of somatic mutations in important cancer-related genes. The vertical red dotted line indicates the boundary of good and bad PDX models according to molecular characteristics. N/A, not available.

Next, we examined how well the mutations identified in patients were reproduced in PDXs. In all 132 samples, it was found that 63% of the exonic mutations in patients were also identified in PDXs on average (Fig. 2A). The portion of common mutations, however, varied tremendously from 0% to 98%. Low rates of common mutations were mostly observed in cases where patient and PDX tumors showed different pathology in histological analysis. Considering 92 cases with consistent histopathological result, 78% of the exonic mutations in patients were also identified in PDXs on average.

We have also examined the overlap of functional mutations, including non-synonymous SNVs, stop-gain mutations, stop-loss mutations, frameshift insertions, and frameshift deletions, between patient and PDX mouse tumors using 517 cancer-related genes curated from OncoKB and other literatures (Fig. 2B) [23-25]. The most commonly mutated gene was *TP53*. For cases with consistent histology, major portion of driver gene mutations were observed commonly in both patient and PDX tumors. Concordant driver genes included many mutations targeted by cancer drugs, including *PIK3CA, EGFR, BRAF*, and *ALK*. Thus, our bank of PDX mouse models can be a useful resource for testing drug efficacy in a preclinical setup.

Then we investigated how the histological analysis results are associated with the rate of common mutations. Out of 119 cases with the histological result available, we had 97 consistent and 22 inconsistent cases (Fig. 2B). The common mutation rates were over 20% in most consistent cases, whereas they were below 3% in most inconsistent cases. Thus, we were able to find 10%–20% of common mutation rate as a general threshold for determining whether the PDX mutations recapitulate the patient’s somatic mutations faithfully (Fig. 2A).

However, there existed several exceptional cases from this guideline. We found only one case where the mutation profiles were concordant, but the histological examination result was different tumors in patient and PDX (Fig. 2B). In contrast, we found nine cases in the opposite direction where the histological examination concluded concordant tumors, but the mutation profiles were vastly different between patient and PDX (Fig. 2B). Reasons for this discrepancy are not clear, but it is difficult to imagine that no common mutation was found if the patient and PDX tumors were truly of the same histology. Histological examination is not a perfect, but error-prone procedure. In conclusion, it is not necessary to compare the mutation profiles if the histological examination result is inconsistent, but when the histological examination gave a good result, comparing mutation profiles can be useful in determining if the PDX tumor is truly identical to the patient tumor. Thus, the mutation profile plays complementary roles to histological examination.

### Mutation and copy number profiles over passages of PDX mouse tumors

The main advantage of PDX tumor model is that it is possible to amplify the amount of tumor cells by engrafting tumor tissue into other immunodeficient mice. Molecular characteristics are usually expected to be preserved in the passage process, but detailed examination at the genome scale is quite rare.

**Fig. 3.**
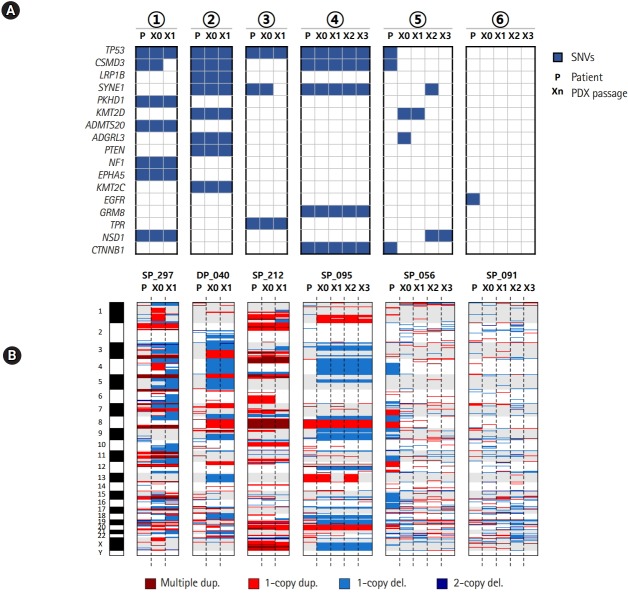
Mutation and copy number profiles over the passage in patient-derived xenograft (PDX) mouse models. (A) Somatic mutations over PDX mouse passages. (B) Copy number profiles over PDX mouse passages. The circled numbers indicating patient cases are consistent with those of Fig. 2B. SNV, single nucleotide variation.

We examined the mutation and copy number profiles for six cases where PDX tumor samples were available over several passages/generations (Fig. 3). We had four histologically consistent cases and two inconsistent cases. Both the mutation and copy number profiles were well reproduced throughout many generations in four good cases. Interestingly, the copy numbers showed much larger changes than somatic mutations especially between patient tumor and the first generation of xenograft tumor (Fig. 3B). It seems that PDX tumors harbor a number of new copy number losses that were maintained over many passages, which again implied that the clonal selection occurred in establishing the PDX mouse tumors.

## Discussion

With the recent advances in anti-cancer drugs from unspecified cytotoxic agents to targeted therapy or immunotherapy, a better preclinical model that reflects the characteristics of each patient is required to realize the precision medicine in cancer. The PDX mouse model has emerged as a valuable preclinical model to overcome the limitations of *in vitro* cell lines. Drug development and targeted therapy studies using PDX models reported that their responses to treatments are consistent with the clinical outcomes of actual patients. However, co-clinical studies are extremely rare and the fidelity of PDX mouse to patient tumors is not well defined.

In this study, we have found that PDX mouse models recapitulate the genetic characteristics of patients quite well. Although it is difficult to assume that PDX models perfectly represent patients' genetic profiles due to the effects of clonal selection or evolution, much of the alterations identified in the patient were identified in PDX tumors as well. In addition, these alterations have been maintained for generations. Importantly, several PDX models have actionable alterations that can help drug development targeting those aberrations.

Although we confirmed that major portion of somatic mutations and copy number alterations were maintained in PDX establishment, we also observed many novel mutations and copy number alterations in PDX mouse tumors. The clonal selection and evolution are the main causes, resulting in different VAFs for several driver mutations and novel copy number losses. We also observed many additional somatic mutations emerge as a result of clonal enrichment. Understanding the details of clonal evolution should be important in interpretation of treatment response using PDX mouse models.

To the best of our knowledge, this is the largest scale of PDX study in lung cancer with matching trio samples. Over 100 cases of PDX biobank data were produced, even though there are cases where the histopathology of the patient and matched PDX are not consistent. By comparing the mutation and copy number profiles of patient and PDX tumors, we were able to show that the molecular characteristics are mostly in agreement with the histological results. But several cases were identified that molecular characteristics did not agree even though the histological examination results were identical in patient and PDX tumors. This highlights the complementary roles of molecular profiling in evaluating the PDX mice as a surrogate model in preclinical tests.

## Figures and Tables

**Table 1.** Clinical information of 132 lung cancer patients

Characteristic	No. (%) (n=132)		
Sex			
Female	44 (34)		
Male	88 (66)		
Age (yr), median	65		
Smoking status			
Nonsmoker	48 (36)		
Smoker	84 (64)		
Clinical stage			
Early stage (I–II)	66 (50)		
Late stage (III–IV)	25 (19)		
N/A	41 (31)		
Subtype			
Adenocarcinoma	54 (41)		
Squamous cell carcinoma	48 (36)		
Large cell carcinoma	4 (3)		
Unclassified	26 (20)		
Recurrent			
Yes	52 (39)		
No	80 (61)		
Metastasis			
Yes	50 (38)		
No	82 (62)		
Death			
Yes	33 (25)		
No	99 (75)		

N/A, not available.

## Supplementary Materials

Supplementary Table 1. Statistics of reads mapping to human reference genome

Supplementary Table 2. Result of Xenome alignment to human and mouse references
