# Supplementary material for: Validity of patient-derived xenograft mouse models for lung cancer based on exome sequencing data
Source: Genomics Inform. 2020 Mar 31;18(1):e3. doi: 10.5808/GI.2020.18.1.e3 (PMC7120347; doi:10.5808/GI.2020.18.1.e3)
Supplement: Supplementary Table 1. — Statistics of reads mapping to human reference genome [file gi-2020-18-1-e3-supple.docx]

**Supplementary Table 1.** Statistics of reads mapping to human reference genome

|  | **Sample** | **Type** | **No. of raw**  **sequence reads** | **No. after trimming** | **No. of paired-end reads** | **No. of mapped reads** | **Mapping rate** |
| --- | --- | --- | --- | --- | --- | --- | --- |
| 1 | DPBL_005 | Normal | 105,112,094 | 94,672,126 | 80,797,328 | 80,761,087 | 99.955 |
| 2 | DPBL_013 | Normal | 89,521,732 | 80,693,341 | 67,895,424 | 67,867,903 | 99.959 |
| 3 | DPBL_014 | Normal | 94,568,070 | 85,177,736 | 72,791,172 | 72,755,396 | 99.951 |
| 4 | DPBL_024 | Normal | 100,657,646 | 89,830,429 | 75,961,918 | 75,924,995 | 99.951 |
| 5 | DPBL_029 | Normal | 95,369,578 | 85,929,492 | 73,409,852 | 73,381,458 | 99.961 |
| 6 | DPBL_031 | Normal | 108,965,562 | 96,923,852 | 81,216,586 | 81,180,011 | 99.955 |
| 7 | DPNT_032 | Normal | 104,570,192 | 93,399,001 | 78,960,222 | 78,918,649 | 99.947 |
| 8 | DPBL_039 | Normal | 104,350,258 | 93,144,160 | 78,763,376 | 78,723,870 | 99.950 |
| 9 | DPBL_040 | Normal | 103,986,334 | 93,629,990 | 78,714,484 | 78,682,063 | 99.959 |
| 10 | DPNT_041 | Normal | 112,514,324 | 101,313,167 | 85,496,658 | 85,461,357 | 99.959 |
| 11 | DPBL_044 | Normal | 114,113,874 | 101,497,010 | 85,063,238 | 85,025,569 | 99.956 |
| 12 | DPNT_048 | Normal | 99,098,862 | 88,390,785 | 74,680,988 | 74,642,092 | 99.948 |
| 13 | DPBL_056 | Normal | 102,141,196 | 91,033,350 | 76,900,112 | 76,861,677 | 99.950 |
| 14 | DPNT_057 | Normal | 110,140,072 | 98,571,261 | 83,473,732 | 83,437,061 | 99.956 |
| 15 | DPNT_060 | Normal | 100,967,228 | 90,305,204 | 76,416,098 | 76,382,041 | 99.955 |
| 16 | DPNT_075 | Normal | 104,446,490 | 93,638,365 | 79,369,594 | 79,306,347 | 99.920 |
| 17 | DPBL_078 | Normal | 98,196,298 | 87,739,540 | 74,258,076 | 74,218,129 | 99.946 |
| 18 | DPNT_081 | Normal | 104,550,408 | 93,435,654 | 79,041,602 | 79,011,224 | 99.962 |
| 19 | DPNT_084 | Normal | 115,214,016 | 103,005,806 | 87,207,462 | 87,167,730 | 99.954 |
| 20 | DPNT_085 | Normal | 95,785,180 | 85,534,070 | 72,225,536 | 72,188,806 | 99.949 |
| 21 | DPNT_088 | Normal | 123,950,120 | 110,812,439 | 94,274,786 | 94,229,484 | 99.952 |
| 22 | DPNT_089 | Normal | 88,158,050 | 78,882,515 | 67,142,952 | 67,114,913 | 99.958 |
| 23 | DPNT_091 | Normal | 104,729,876 | 93,747,677 | 79,785,908 | 79,749,967 | 99.955 |
| 24 | DPNT_094 | Normal | 104,099,872 | 93,143,488 | 79,241,402 | 79,200,645 | 99.949 |
| 25 | SPNT_008 | Normal | 79,045,888 | 70,907,665 | 59,639,700 | 59,608,493 | 99.948 |
| 26 | SPNT_012 | Normal | 52,879,668 | 47,213,326 | 39,539,046 | 39,520,446 | 99.953 |
| 27 | SPNT_013 | Normal | 61,909,590 | 55,259,624 | 46,245,504 | 46,220,205 | 99.945 |
| 28 | SPNT_023 | Normal | 83,340,612 | 74,958,237 | 64,044,534 | 64,011,335 | 99.948 |
| 29 | SPNT_029 | Normal | 61,073,048 | 54,645,889 | 45,836,306 | 45,811,800 | 99.947 |
| 30 | SPNT_036 | Normal | 90,829,250 | 81,416,056 | 69,307,224 | 69,269,108 | 99.945 |
| 31 | SPNT_041 | Normal | 58,249,098 | 52,116,364 | 43,709,674 | 43,689,575 | 99.954 |
| 32 | SPNT_044 | Normal | 80,185,996 | 72,182,227 | 60,795,356 | 60,764,123 | 99.949 |
| 33 | SPNT_055 | Normal | 80,018,962 | 71,832,848 | 60,484,252 | 60,457,461 | 99.956 |
| 34 | SPNT_056 | Normal | 74,484,454 | 67,034,712 | 56,440,778 | 56,410,581 | 99.946 |
| 35 | SPNT_060 | Normal | 66,940,564 | 59,966,701 | 50,413,404 | 50,386,296 | 99.946 |
| 36 | SPNT_061 | Normal | 72,872,894 | 65,360,956 | 55,041,334 | 55,015,397 | 99.953 |
| 37 | SPNT_071 | Normal | 75,346,018 | 67,711,404 | 56,952,744 | 56,922,232 | 99.946 |
| 38 | SPNT_073 | Normal | 74,447,822 | 66,808,942 | 56,153,204 | 56,121,416 | 99.943 |
| 39 | SPNT_079 | Normal | 73,399,362 | 65,950,504 | 55,632,928 | 55,608,358 | 99.956 |
| 40 | SPNT_082 | Normal | 85,899,606 | 77,225,900 | 65,987,194 | 65,951,133 | 99.945 |
| 41 | SPNT_091 | Normal | 88,885,104 | 79,446,270 | 67,726,224 | 67,673,979 | 99.923 |
| 42 | SPNT_095 | Normal | 77,261,412 | 69,332,904 | 58,402,596 | 58,366,721 | 99.939 |
| 43 | SPNT_100 | Normal | 71,919,414 | 64,630,672 | 54,476,806 | 54,448,791 | 99.949 |
| 44 | SPNT_115 | Normal | 71,941,850 | 64,550,601 | 54,330,706 | 54,297,293 | 99.939 |
| 45 | SPBL_163 | Normal | 81,164,794 | 72,267,765 | 60,699,546 | 60,628,540 | 99.883 |
| 46 | SPBL_173 | Normal | 96,180,546 | 85,589,530 | 71,833,030 | 71,778,094 | 99.924 |
| 47 | SPBL_182 | Normal | 91,582,732 | 82,133,988 | 69,996,620 | 69,963,859 | 99.953 |
| 48 | SPNT_184 | Normal | 102,639,460 | 91,990,440 | 78,425,912 | 78,386,124 | 99.949 |
| 49 | SPNT_185 | Normal | 98,196,874 | 87,364,992 | 72,899,778 | 72,830,923 | 99.906 |
| 50 | SPBL_186 | Normal | 100,888,886 | 90,655,097 | 76,781,986 | 76,730,897 | 99.933 |
| 51 | SPBL_190 | Normal | 86,832,496 | 78,050,298 | 66,094,832 | 66,047,014 | 99.928 |
| 52 | SPBL_191 | Normal | 89,452,682 | 80,491,051 | 68,173,940 | 68,135,021 | 99.943 |
| 53 | SPBL_193 | Normal | 86,876,126 | 78,140,462 | 66,210,012 | 66,157,932 | 99.921 |
| 54 | SPNT_197 | Normal | 93,282,770 | 83,208,533 | 69,626,958 | 69,570,143 | 99.918 |
| 55 | SPBL_198 | Normal | 90,977,274 | 81,715,770 | 69,122,732 | 69,074,656 | 99.930 |
| 56 | SPBL_201 | Normal | 122,330,792 | 110,441,392 | 93,197,626 | 93,165,058 | 99.965 |
| 57 | SPNT_203 | Normal | 133,996,046 | 120,096,101 | 102,308,962 | 102,261,950 | 99.954 |
| 58 | SPNT_205 | Normal | 109,949,866 | 98,904,289 | 83,383,896 | 83,349,996 | 99.959 |
| 59 | SPNT_206 | Normal | 102,673,258 | 92,220,033 | 78,164,514 | 78,129,907 | 99.956 |
| 60 | SPNT_207 | Normal | 89,432,888 | 80,160,373 | 68,327,480 | 68,295,570 | 99.953 |
| 61 | SPNT_208 | Normal | 95,772,886 | 85,745,811 | 72,876,912 | 72,839,106 | 99.948 |
| 62 | SPNT_211 | Normal | 106,017,164 | 95,148,099 | 80,527,800 | 80,494,561 | 99.959 |
| 63 | SPNT_212 | Normal | 92,223,378 | 82,170,252 | 68,682,798 | 68,622,156 | 99.912 |
| 64 | SPNT_213 | Normal | 107,265,266 | 96,139,795 | 81,427,510 | 81,391,852 | 99.956 |
| 65 | SPNT_214 | Normal | 95,114,052 | 84,717,238 | 70,775,714 | 70,711,455 | 99.909 |
| 66 | SPNT_215 | Normal | 113,333,256 | 101,912,269 | 86,404,208 | 86,371,410 | 99.962 |
| 67 | SPNT_216 | Normal | 110,895,082 | 99,305,738 | 84,740,062 | 84,703,469 | 99.957 |
| 68 | SPNT_217 | Normal | 104,294,212 | 93,763,624 | 79,499,894 | 79,471,019 | 99.964 |
| 69 | SPNT_218 | Normal | 103,297,414 | 92,740,530 | 78,721,846 | 78,684,678 | 99.953 |
| 70 | SPNT_220 | Normal | 87,668,574 | 77,948,754 | 65,350,368 | 65,287,511 | 99.904 |
| 71 | SPNT_222 | Normal | 102,423,614 | 91,888,485 | 77,704,890 | 77,672,871 | 99.959 |
| 72 | SPBL_223 | Normal | 97,856,970 | 80,795,590 | 61,987,938 | 61,958,391 | 99.952 |
| 73 | SPNT_231 | Normal | 94,603,444 | 84,055,400 | 69,995,810 | 69,921,782 | 99.894 |
| 74 | SPNT_235 | Normal | 90,065,794 | 80,293,006 | 67,434,748 | 67,393,859 | 99.939 |
| 75 | SPBL_238 | Normal | 113,649,704 | 102,097,770 | 86,709,886 | 86,672,198 | 99.957 |
| 76 | SPBL_241 | Normal | 99,958,142 | 82,553,390 | 63,298,356 | 63,268,347 | 99.953 |
| 77 | SPNT_244 | Normal | 102,544,350 | 91,946,048 | 77,751,454 | 77,716,927 | 99.956 |
| 78 | SPBL_252 | Normal | 94,786,950 | 78,293,230 | 60,061,854 | 60,034,190 | 99.954 |
| 79 | SPBL_260 | Normal | 88,715,768 | 79,646,415 | 67,385,078 | 67,342,729 | 99.937 |
| 80 | SPBL_262 | Normal | 103,385,350 | 85,822,346 | 66,088,124 | 66,055,791 | 99.951 |
| 81 | SPNT_265 | Normal | 121,090,816 | 108,807,102 | 92,413,298 | 92,370,800 | 99.954 |
| 82 | SPBL_269 | Normal | 115,819,286 | 95,512,049 | 73,116,228 | 73,086,642 | 99.960 |
| 83 | SPBL_271 | Normal | 92,477,270 | 76,726,461 | 59,154,638 | 59,126,453 | 99.952 |
| 84 | SPNT_276 | Normal | 107,816,494 | 96,883,812 | 81,950,616 | 81,920,151 | 99.963 |
| 85 | SPBL_278 | Normal | 90,121,954 | 74,795,292 | 57,593,918 | 57,564,515 | 99.949 |
| 86 | SPNT_281 | Normal | 92,671,060 | 83,342,929 | 70,846,132 | 70,817,849 | 99.960 |
| 87 | SPNT_284 | Normal | 119,939,196 | 107,784,217 | 90,710,256 | 90,671,507 | 99.957 |
| 88 | SPBL_285 | Normal | 96,808,590 | 80,439,788 | 61,977,684 | 61,942,966 | 99.944 |
| 89 | SPNT_289 | Normal | 103,929,482 | 93,397,992 | 79,078,798 | 79,049,596 | 99.963 |
| 90 | SPNT_290 | Normal | 105,196,306 | 94,405,559 | 79,790,630 | 79,755,365 | 99.956 |
| 91 | SPBL_292 | Normal | 101,403,796 | 91,026,882 | 77,270,952 | 77,235,823 | 99.955 |
| 92 | SPNT_293 | Normal | 103,880,264 | 93,252,853 | 78,989,114 | 78,956,442 | 99.959 |
| 93 | SPNT_295 | Normal | 107,107,790 | 96,153,176 | 81,308,640 | 81,270,932 | 99.954 |
| 94 | SPNT_297 | Normal | 97,148,852 | 86,360,468 | 72,381,948 | 72,094,580 | 99.603 |
| 95 | SPNT_299 | Normal | 115,881,894 | 103,756,742 | 88,432,370 | 88,399,222 | 99.963 |
| 96 | SPNT_302 | Normal | 104,261,286 | 86,169,627 | 66,065,942 | 66,039,661 | 99.960 |
| 97 | SPBL_307 | Normal | 102,466,284 | 92,334,778 | 77,921,288 | 77,886,663 | 99.956 |
| 98 | SPNT_310 | Normal | 102,795,016 | 92,162,245 | 77,975,108 | 77,901,829 | 99.906 |
| 99 | SPBL_318 | Normal | 105,006,404 | 94,649,373 | 79,867,086 | 79,832,016 | 99.956 |
| 100 | SPBL_341 | Normal | 113,559,870 | 102,559,198 | 86,570,376 | 86,538,103 | 99.963 |
| 101 | SPNT_347 | Normal | 115,733,630 | 104,003,308 | 88,162,526 | 88,126,566 | 99.959 |
| 102 | SPBL_353 | Normal | 107,246,340 | 96,533,539 | 81,380,048 | 81,347,681 | 99.960 |
| 103 | SPBL_363 | Normal | 103,168,848 | 92,177,000 | 78,400,142 | 78,359,343 | 99.948 |
| 104 | SPBL_364 | Normal | 121,253,924 | 109,549,184 | 92,651,890 | 92,614,407 | 99.960 |
| 105 | SPBL_365 | Normal | 90,001,446 | 80,426,187 | 68,454,444 | 68,419,552 | 99.949 |
| 106 | SPNT_366 | Normal | 79,734,310 | 71,271,456 | 60,602,796 | 60,575,381 | 99.955 |
| 107 | SPNT_367 | Normal | 108,017,602 | 97,161,701 | 82,563,504 | 82,524,809 | 99.953 |
| 108 | SPNT_369 | Normal | 123,442,274 | 102,020,127 | 78,231,390 | 78,200,514 | 99.961 |
| 109 | SPNT_372 | Normal | 97,387,428 | 86,860,921 | 73,867,062 | 73,834,423 | 99.956 |
| 110 | SPBL_377 | Normal | 93,706,830 | 83,640,468 | 71,152,988 | 71,111,375 | 99.942 |
| 111 | SPNT_383 | Normal | 98,761,786 | 88,187,049 | 74,988,574 | 74,950,840 | 99.950 |
| 112 | SPNT_402 | Normal | 98,185,018 | 87,664,443 | 74,595,628 | 74,564,394 | 99.958 |
| 113 | SPNT_409 | Normal | 95,642,206 | 85,252,425 | 72,485,634 | 72,452,276 | 99.954 |
| 114 | SPNT_410 | Normal | 96,617,592 | 86,323,552 | 73,513,132 | 73,479,987 | 99.955 |
| 115 | SPNT_415 | Normal | 94,269,942 | 84,201,086 | 71,705,028 | 71,673,320 | 99.956 |
| 116 | SPNT_416 | Normal | 100,864,402 | 89,954,032 | 76,553,478 | 76,516,694 | 99.952 |
| 117 | SPNT_417 | Normal | 101,292,288 | 90,314,497 | 76,781,896 | 76,745,896 | 99.953 |
| 118 | SPBL_418 | Normal | 98,071,074 | 87,443,587 | 74,401,096 | 74,367,718 | 99.955 |
| 119 | SPBL_419 | Normal | 85,733,934 | 76,415,346 | 64,877,434 | 64,841,776 | 99.945 |
| 120 | SPBL_420 | Normal | 96,873,438 | 86,254,226 | 73,080,870 | 73,046,644 | 99.953 |
| 121 | SPNT_428 | Normal | 92,696,092 | 82,768,302 | 70,341,526 | 70,311,373 | 99.957 |
| 122 | SPNT_434 | Normal | 99,952,000 | 89,202,115 | 75,666,050 | 75,627,955 | 99.950 |
| 123 | SPNT_435 | Normal | 88,531,932 | 79,091,774 | 67,232,738 | 67,207,064 | 99.962 |
| 124 | SPNT_436 | Normal | 99,830,530 | 89,041,452 | 75,556,302 | 75,526,046 | 99.960 |
| 125 | SPBL_441 | Normal | 102,811,602 | 91,557,920 | 77,619,204 | 77,579,728 | 99.949 |
| 126 | SPNT_451 | Normal | 116,934,658 | 104,520,376 | 88,202,304 | 88,159,129 | 99.951 |
| 127 | SPNT_454 | Normal | 96,849,038 | 86,555,821 | 73,061,982 | 73,030,154 | 99.956 |
| 128 | SPNT_464 | Normal | 113,442,950 | 101,206,632 | 85,318,976 | 85,268,523 | 99.941 |
| 129 | SPBL_467 | Normal | 106,616,914 | 95,097,490 | 80,120,008 | 80,081,623 | 99.952 |
| 130 | SPBL_473 | Normal | 103,935,820 | 92,723,474 | 78,173,040 | 78,131,889 | 99.947 |
| 131 | SPNT_480 | Normal | 93,879,530 | 83,762,366 | 70,513,798 | 70,481,623 | 99.954 |
| 132 | SPNT_482 | Normal | 107,043,260 | 95,458,163 | 80,673,812 | 80,636,641 | 99.954 |
| 133 | DPTT_005 | Tumor | 98,444,818 | 88,897,541 | 75,215,858 | 75,183,977 | 99.958 |
| 134 | DPTT_013 | Tumor | 99,933,688 | 89,994,535 | 75,911,654 | 75,874,194 | 99.951 |
| 135 | DPTT_014 | Tumor | 98,053,584 | 88,573,787 | 74,957,858 | 74,927,208 | 99.959 |
| 136 | DPTT_024 | Tumor | 109,148,986 | 97,463,633 | 82,881,228 | 82,833,573 | 99.943 |
| 137 | DPTT_029 | Tumor | 106,708,046 | 96,151,587 | 81,239,720 | 81,212,942 | 99.967 |
| 138 | DPTT_031 | Tumor | 117,679,120 | 106,003,192 | 89,413,412 | 89,375,960 | 99.958 |
| 139 | DPTT_032 | Tumor | 106,422,708 | 95,052,285 | 80,888,732 | 80,851,130 | 99.954 |
| 140 | DPTT_039 | Tumor | 96,794,144 | 86,531,212 | 73,652,064 | 73,621,609 | 99.959 |
| 141 | DPTT_040 | Tumor | 108,390,156 | 97,578,080 | 82,282,822 | 82,250,131 | 99.960 |
| 142 | DPTT_041 | Tumor | 111,976,988 | 99,480,590 | 83,283,312 | 83,247,033 | 99.956 |
| 143 | DPTT_044 | Tumor | 100,432,760 | 89,334,081 | 74,916,340 | 74,884,352 | 99.957 |
| 144 | DPTT_048 | Tumor | 99,212,412 | 88,668,562 | 75,526,242 | 75,491,223 | 99.954 |
| 145 | DPTT_056 | Tumor | 105,041,596 | 93,830,217 | 79,817,552 | 79,783,366 | 99.957 |
| 146 | DPTT_057 | Tumor | 104,151,634 | 93,343,976 | 79,129,158 | 79,096,485 | 99.959 |
| 147 | DPTT_060 | Tumor | 104,701,588 | 93,527,416 | 79,616,726 | 79,580,000 | 99.954 |
| 148 | DPTT_075 | Tumor | 103,707,498 | 92,498,990 | 78,454,776 | 78,414,294 | 99.948 |
| 149 | DPTT_078 | Tumor | 95,233,524 | 85,422,881 | 72,556,912 | 72,527,650 | 99.960 |
| 150 | DPTT_081 | Tumor | 105,955,694 | 94,209,605 | 79,439,816 | 79,397,961 | 99.947 |
| 151 | DPTT_084 | Tumor | 96,920,088 | 86,041,666 | 72,426,516 | 72,378,822 | 99.934 |
| 152 | DPTT_085 | Tumor | 103,467,966 | 92,165,278 | 77,829,472 | 77,791,007 | 99.951 |
| 153 | DPTT_088 | Tumor | 87,434,660 | 77,860,229 | 65,713,868 | 65,681,164 | 99.950 |
| 154 | DPTT_089 | Tumor | 99,686,110 | 88,465,781 | 74,394,546 | 74,348,104 | 99.938 |
| 155 | DPTT_091 | Tumor | 96,011,846 | 85,479,401 | 72,215,128 | 72,183,175 | 99.956 |
| 156 | DPTT_094 | Tumor | 103,548,808 | 92,628,679 | 78,656,640 | 78,620,565 | 99.954 |
| 157 | SPTT_008 | Tumor | 80,785,964 | 72,385,487 | 60,797,320 | 60,759,386 | 99.938 |
| 158 | SPTT_012 | Tumor | 60,211,972 | 53,944,555 | 45,533,176 | 45,508,905 | 99.947 |
| 159 | SPTT_013 | Tumor | 55,995,112 | 50,112,874 | 42,228,656 | 42,205,203 | 99.944 |
| 160 | SPTT_023 | Tumor | 74,810,946 | 66,886,678 | 57,095,610 | 57,061,689 | 99.941 |
| 161 | SPTT_029 | Tumor | 64,532,784 | 57,747,516 | 48,470,726 | 48,449,144 | 99.955 |
| 162 | SPTT_036 | Tumor | 98,048,934 | 87,577,399 | 74,398,608 | 74,347,318 | 99.931 |
| 163 | SPTT_041 | Tumor | 58,045,258 | 51,856,840 | 43,436,492 | 43,418,541 | 99.959 |
| 164 | SPTT_044 | Tumor | 68,871,388 | 61,588,750 | 51,645,026 | 51,618,506 | 99.949 |
| 165 | SPTT_055 | Tumor | 82,318,710 | 73,884,593 | 62,251,130 | 62,227,010 | 99.961 |
| 166 | SPTT_056 | Tumor | 61,151,634 | 54,790,317 | 46,040,954 | 46,019,128 | 99.953 |
| 167 | SPTT_060 | Tumor | 71,763,262 | 64,348,005 | 54,171,604 | 54,143,536 | 99.948 |
| 168 | SPTT_061 | Tumor | 77,078,170 | 69,075,143 | 58,051,820 | 58,023,695 | 99.952 |
| 169 | SPTT_071 | Tumor | 65,584,228 | 58,714,420 | 49,276,354 | 49,254,323 | 99.955 |
| 170 | SPTT_073 | Tumor | 56,711,818 | 50,718,201 | 42,526,396 | 42,507,179 | 99.955 |
| 171 | SPTT_079 | Tumor | 90,668,354 | 81,435,000 | 68,623,426 | 68,592,680 | 99.955 |
| 172 | SPTT_082 | Tumor | 98,719,270 | 88,692,809 | 76,131,452 | 76,077,483 | 99.929 |
| 173 | SPTT_091 | Tumor | 83,589,594 | 75,138,627 | 64,201,616 | 64,162,987 | 99.940 |
| 174 | SPTT_095 | Tumor | 75,716,450 | 67,875,440 | 57,109,900 | 57,073,265 | 99.936 |
| 175 | SPTT_100 | Tumor | 76,958,560 | 69,140,644 | 58,255,656 | 58,227,154 | 99.951 |
| 176 | SPTT_115 | Tumor | 73,117,708 | 65,610,045 | 55,261,762 | 55,232,420 | 99.947 |
| 177 | SPTT_163 | Tumor | 78,694,128 | 70,463,780 | 59,756,298 | 59,712,337 | 99.926 |
| 178 | SPTT_173 | Tumor | 104,786,476 | 93,727,090 | 79,448,064 | 79,396,492 | 99.935 |
| 179 | SPTT_182 | Tumor | 103,533,678 | 92,603,970 | 78,884,878 | 78,846,505 | 99.951 |
| 180 | SPTT_184 | Tumor | 95,870,122 | 85,709,734 | 72,801,508 | 72,764,125 | 99.949 |
| 181 | SPTT_185 | Tumor | 78,954,286 | 70,591,994 | 59,840,556 | 59,793,838 | 99.922 |
| 182 | SPTT_186 | Tumor | 90,530,732 | 80,966,795 | 68,652,868 | 68,302,946 | 99.490 |
| 183 | SPTT_190 | Tumor | 80,012,916 | 71,486,691 | 60,570,100 | 60,512,996 | 99.906 |
| 184 | SPTT_191 | Tumor | 98,578,466 | 87,942,664 | 74,271,424 | 74,212,205 | 99.920 |
| 185 | SPTT_193 | Tumor | 86,363,580 | 71,676,148 | 55,188,086 | 55,161,042 | 99.951 |
| 186 | SPTT_197 | Tumor | 91,858,410 | 82,031,968 | 69,454,518 | 69,401,005 | 99.923 |
| 187 | SPTT_198 | Tumor | 92,339,592 | 82,546,138 | 69,894,450 | 69,850,252 | 99.937 |
| 188 | SPTT_201 | Tumor | 111,992,898 | 100,420,743 | 84,723,374 | 84,683,995 | 99.954 |
| 189 | SPTT_203 | Tumor | 104,320,420 | 93,369,169 | 79,270,058 | 79,230,379 | 99.950 |
| 190 | SPTT_205 | Tumor | 106,420,220 | 94,759,094 | 79,495,214 | 79,457,245 | 99.952 |
| 191 | SPTT_206 | Tumor | 98,250,830 | 87,151,396 | 72,876,398 | 72,830,514 | 99.937 |
| 192 | SPTT_207 | Tumor | 111,684,488 | 100,070,445 | 85,003,554 | 84,964,758 | 99.954 |
| 193 | SPTT_208 | Tumor | 86,548,284 | 77,634,391 | 65,981,528 | 65,955,498 | 99.961 |
| 194 | SPTT_211 | Tumor | 94,379,176 | 84,749,556 | 72,015,310 | 71,976,690 | 99.946 |
| 195 | SPTT_212 | Tumor | 92,759,628 | 82,918,816 | 70,262,452 | 70,210,499 | 99.926 |
| 196 | SPTT_213 | Tumor | 107,154,188 | 95,927,499 | 81,472,642 | 81,437,087 | 99.956 |
| 197 | SPTT_214 | Tumor | 90,984,530 | 81,354,416 | 68,940,242 | 68,893,637 | 99.932 |
| 198 | SPTT_215 | Tumor | 95,821,954 | 86,086,947 | 72,799,730 | 72,762,105 | 99.948 |
| 199 | SPTT_216 | Tumor | 110,937,948 | 99,557,526 | 84,071,906 | 84,004,279 | 99.920 |
| 200 | SPTT_217 | Tumor | 98,652,320 | 88,619,621 | 74,900,592 | 74,861,135 | 99.947 |
| 201 | SPTT_218 | Tumor | 90,248,256 | 80,795,307 | 68,538,668 | 68,498,090 | 99.941 |
| 202 | SPTT_220 | Tumor | 96,120,082 | 85,829,400 | 72,807,840 | 72,731,474 | 99.895 |
| 203 | SPTT_222 | Tumor | 99,592,082 | 89,367,412 | 75,462,170 | 75,424,588 | 99.950 |
| 204 | SPTT_223 | Tumor | 108,382,748 | 96,868,543 | 82,087,942 | 82,047,045 | 99.950 |
| 205 | SPTT_231 | Tumor | 96,730,236 | 80,267,556 | 61,754,150 | 61,698,116 | 99.909 |
| 206 | SPTT_235 | Tumor | 93,516,922 | 83,498,402 | 70,682,588 | 70,629,471 | 99.925 |
| 207 | SPTT_238 | Tumor | 107,242,122 | 96,025,023 | 81,499,274 | 81,465,810 | 99.959 |
| 208 | SPTT_241 | Tumor | 109,282,360 | 98,155,383 | 83,490,502 | 83,438,496 | 99.938 |
| 209 | SPTT_244 | Tumor | 117,440,038 | 105,603,554 | 89,411,724 | 89,365,809 | 99.949 |
| 210 | SPTT_252 | Tumor | 99,657,718 | 89,709,447 | 76,101,512 | 76,064,974 | 99.952 |
| 211 | SPTT_260 | Tumor | 98,873,624 | 88,153,215 | 74,502,772 | 74,456,705 | 99.938 |
| 212 | SPTT_262 | Tumor | 93,909,962 | 83,928,066 | 71,293,572 | 71,232,299 | 99.914 |
| 213 | SPTT_265 | Tumor | 102,871,920 | 92,120,825 | 78,562,092 | 78,517,107 | 99.943 |
| 214 | SPTT_269 | Tumor | 98,777,688 | 88,695,933 | 75,054,154 | 75,018,253 | 99.952 |
| 215 | SPTT_271 | Tumor | 85,876,044 | 76,651,039 | 65,054,314 | 64,739,492 | 99.516 |
| 216 | SPTT_276 | Tumor | 87,952,458 | 79,058,318 | 66,986,910 | 66,962,397 | 99.963 |
| 217 | SPTT_278 | Tumor | 92,293,410 | 82,500,567 | 70,054,606 | 69,994,796 | 99.915 |
| 218 | SPTT_281 | Tumor | 85,840,776 | 76,966,739 | 65,777,054 | 65,752,013 | 99.962 |
| 219 | SPTT_284 | Tumor | 97,869,800 | 87,828,650 | 74,610,136 | 74,571,644 | 99.948 |
| 220 | SPTT_285 | Tumor | 91,698,926 | 82,030,847 | 69,673,790 | 69,624,948 | 99.930 |
| 221 | SPTT_289 | Tumor | 100,086,270 | 89,851,261 | 75,508,292 | 75,479,776 | 99.962 |
| 222 | SPTT_290 | Tumor | 88,552,530 | 79,390,377 | 67,374,518 | 67,335,573 | 99.942 |
| 223 | SPTT_292 | Tumor | 137,084,366 | 122,502,012 | 104,355,046 | 104,305,167 | 99.952 |
| 224 | SPTT_293 | Tumor | 92,778,204 | 83,812,804 | 70,741,368 | 70,711,224 | 99.957 |
| 225 | SPTT_295 | Tumor | 109,567,512 | 98,332,004 | 83,468,840 | 83,428,265 | 99.951 |
| 226 | SPTT_297 | Tumor | 88,684,030 | 79,034,047 | 66,048,564 | 66,008,798 | 99.940 |
| 227 | SPTT_299 | Tumor | 106,219,566 | 95,158,949 | 81,260,514 | 81,228,428 | 99.961 |
| 228 | SPTT_302 | Tumor | 108,086,788 | 96,839,132 | 82,323,690 | 82,293,027 | 99.963 |
| 229 | SPTT_307 | Tumor | 102,892,944 | 92,045,021 | 78,106,142 | 78,073,591 | 99.958 |
| 230 | SPTT_310 | Tumor | 96,302,166 | 86,325,031 | 73,077,110 | 73,050,337 | 99.963 |
| 231 | SPTT_318 | Tumor | 98,822,442 | 88,959,716 | 75,034,986 | 75,004,610 | 99.960 |
| 232 | SPTT_341 | Tumor | 99,180,492 | 89,235,324 | 75,657,506 | 75,626,044 | 99.958 |
| 233 | SPTT_347 | Tumor | 105,395,770 | 94,612,128 | 80,218,672 | 80,185,633 | 99.959 |
| 234 | SPTT_353 | Tumor | 107,126,864 | 96,493,165 | 81,863,008 | 81,827,512 | 99.957 |
| 235 | SPTT_363 | Tumor | 114,808,136 | 102,874,458 | 87,309,410 | 87,267,341 | 99.952 |
| 236 | SPTT_364 | Tumor | 97,915,842 | 88,093,615 | 74,662,330 | 74,630,491 | 99.957 |
| 237 | SPTT_365 | Tumor | 95,525,140 | 85,676,749 | 72,781,610 | 72,748,830 | 99.955 |
| 238 | SPTT_366 | Tumor | 106,108,368 | 95,009,297 | 80,545,244 | 80,508,039 | 99.954 |
| 239 | SPTT_367 | Tumor | 106,611,702 | 95,795,983 | 81,264,164 | 81,227,026 | 99.954 |
| 240 | SPTT_369 | Tumor | 116,672,672 | 104,956,787 | 89,009,170 | 88,974,115 | 99.961 |
| 241 | SPTT_372 | Tumor | 94,553,010 | 84,834,262 | 72,071,648 | 72,039,763 | 99.956 |
| 242 | SPTT_377 | Tumor | 94,497,636 | 84,484,937 | 71,507,268 | 71,464,461 | 99.940 |
| 243 | SPTT_383 | Tumor | 123,836,304 | 109,297,233 | 92,823,918 | 92,779,303 | 99.952 |
| 244 | SPTT_402 | Tumor | 92,479,107 | 80,870,931 | 68,876,358 | 68,847,012 | 99.957 |
| 245 | SPTT_409 | Tumor | 106,005,897 | 93,990,435 | 79,971,314 | 79,943,969 | 99.966 |
| 246 | SPTT_410 | Tumor | 97,251,316 | 87,192,308 | 74,234,716 | 74,199,986 | 99.953 |
| 247 | SPTT_415 | Tumor | 104,787,236 | 93,926,996 | 79,905,544 | 79,870,592 | 99.956 |
| 248 | SPTT_416 | Tumor | 99,940,820 | 89,224,961 | 75,578,104 | 75,506,889 | 99.906 |
| 249 | SPTT_417 | Tumor | 101,892,740 | 91,210,282 | 77,366,366 | 77,330,953 | 99.954 |
| 250 | SPTT_418 | Tumor | 106,326,778 | 95,114,166 | 81,177,518 | 81,135,272 | 99.948 |
| 251 | SPTT_419 | Tumor | 99,400,908 | 89,083,432 | 76,186,140 | 76,146,313 | 99.948 |
| 252 | SPTT_420 | Tumor | 119,806,000 | 107,232,750 | 91,528,518 | 91,482,772 | 99.950 |
| 253 | SPTT_428 | Tumor | 104,076,986 | 93,325,271 | 79,830,686 | 79,788,227 | 99.947 |
| 254 | SPTT_434 | Tumor | 104,906,354 | 93,834,221 | 80,048,220 | 80,010,091 | 99.952 |
| 255 | SPTT_435 | Tumor | 109,484,434 | 97,958,179 | 83,519,972 | 83,481,344 | 99.954 |
| 256 | SPTT_436 | Tumor | 98,526,664 | 88,113,531 | 75,119,194 | 75,089,259 | 99.960 |
| 257 | SPTT_441 | Tumor | 101,698,980 | 90,911,536 | 77,547,560 | 77,515,825 | 99.959 |
| 258 | SPTT_451 | Tumor | 98,593,280 | 88,129,372 | 75,110,082 | 75,074,842 | 99.953 |
| 259 | SPTT_454 | Tumor | 82,746,790 | 74,064,773 | 62,664,852 | 62,634,120 | 99.951 |
| 260 | SPTT_464 | Tumor | 90,507,910 | 81,199,508 | 68,883,600 | 68,855,343 | 99.959 |
| 261 | SPTT_467 | Tumor | 97,464,016 | 87,095,957 | 74,255,078 | 74,223,193 | 99.957 |
| 262 | SPTT_473 | Tumor | 100,403,626 | 89,557,038 | 76,059,830 | 76,022,092 | 99.950 |
| 263 | SPTT_480 | Tumor | 90,049,490 | 79,691,343 | 67,150,808 | 67,038,067 | 99.832 |
| 264 | SPTT_482 | Tumor | 104,731,816 | 93,522,740 | 79,586,834 | 79,550,740 | 99.955 |
| 265 | DPX0_005 | PDX | 158,532,280 | 138,146,594 | 117,581,012 | 117,572,603 | 99.993 |
| 266 | DPX0_013 | PDX | 160,168,216 | 133,465,891 | 108,206,626 | 108,200,750 | 99.995 |
| 267 | DPX0_014 | PDX | 121,352,434 | 106,122,778 | 78,386,816 | 78,376,239 | 99.987 |
| 268 | DPX0_024 | PDX | 142,019,820 | 123,406,892 | 94,830,892 | 94,818,844 | 99.987 |
| 269 | DPX1_029 | PDX | 156,496,100 | 136,959,267 | 106,689,166 | 106,681,818 | 99.993 |
| 270 | DPX0_031 | PDX | 114,521,548 | 99,753,052 | 84,203,864 | 84,192,852 | 99.987 |
| 271 | DPX0_032 | PDX | 117,276,054 | 102,148,882 | 79,953,634 | 79,939,888 | 99.983 |
| 272 | DPX0_039 | PDX | 123,998,480 | 107,857,591 | 86,796,316 | 86,786,750 | 99.989 |
| 273 | DPX0_040 | PDX | 121,054,368 | 105,348,820 | 81,353,712 | 81,341,040 | 99.984 |
| 274 | DPX0_041 | PDX | 124,721,922 | 108,493,702 | 75,783,898 | 75,770,154 | 99.982 |
| 275 | DPX0_044 | PDX | 142,644,454 | 124,713,632 | 103,103,602 | 103,089,325 | 99.986 |
| 276 | DPX0_048 | PDX | 129,719,462 | 113,349,930 | 91,970,368 | 91,955,298 | 99.984 |
| 277 | DPX0_056 | PDX | 190,707,554 | 166,592,535 | 144,109,156 | 144,098,289 | 99.992 |
| 278 | DPX0_057 | PDX | 185,550,048 | 162,194,800 | 140,268,278 | 140,257,265 | 99.992 |
| 279 | DPX0_060 | PDX | 135,842,194 | 118,895,485 | 98,109,542 | 98,098,951 | 99.989 |
| 280 | DPX0_075 | PDX | 128,228,466 | 111,756,420 | 92,163,852 | 92,150,824 | 99.986 |
| 281 | DPX0_078 | PDX | 132,553,482 | 115,716,437 | 80,035,956 | 80,022,178 | 99.983 |
| 282 | DPX0_081 | PDX | 135,918,194 | 118,557,383 | 93,691,298 | 93,678,207 | 99.986 |
| 283 | DPX0_084 | PDX | 137,363,032 | 119,938,231 | 99,401,782 | 99,387,528 | 99.986 |
| 284 | DPX0_085 | PDX | 141,629,480 | 123,495,111 | 99,718,182 | 99,704,830 | 99.987 |
| 285 | DPX0_088 | PDX | 148,588,290 | 129,721,719 | 108,922,628 | 108,908,034 | 99.987 |
| 286 | DPX0_089 | PDX | 142,143,454 | 123,766,379 | 105,547,530 | 105,532,344 | 99.986 |
| 287 | DPX0_091 | PDX | 122,934,718 | 107,223,208 | 87,416,140 | 87,405,491 | 99.988 |
| 288 | DPX0_094 | PDX | 134,231,932 | 116,851,348 | 87,175,532 | 87,164,736 | 99.988 |
| 289 | SPX0_008 | PDX | 104,460,536 | 91,624,080 | 75,284,940 | 75,276,766 | 99.989 |
| 290 | SPX0_012 | PDX | 66,193,112 | 57,389,149 | 48,156,308 | 48,150,367 | 99.988 |
| 291 | SPX0_013 | PDX | 65,019,046 | 56,444,268 | 47,207,738 | 47,201,486 | 99.987 |
| 292 | SPX0_023 | PDX | 86,957,440 | 76,061,457 | 62,538,178 | 62,529,525 | 99.986 |
| 293 | SPX0_029 | PDX | 53,121,380 | 46,115,767 | 38,119,318 | 38,115,187 | 99.989 |
| 294 | SPX1_036 | PDX | 140,856,146 | 122,077,288 | 103,819,320 | 103,805,338 | 99.987 |
| 295 | SPX0_041 | PDX | 63,788,676 | 55,372,093 | 46,704,086 | 46,699,022 | 99.989 |
| 296 | SPX0_044 | PDX | 62,592,958 | 54,256,062 | 46,068,336 | 46,062,429 | 99.987 |
| 297 | SPX0_055 | PDX | 74,260,488 | 65,129,418 | 43,484,540 | 43,480,112 | 99.990 |
| 298 | SPX0_056 | PDX | 62,644,466 | 54,356,390 | 44,369,244 | 44,363,376 | 99.987 |
| 299 | SPX0_060 | PDX | 77,782,110 | 68,225,840 | 49,582,706 | 49,576,952 | 99.988 |
| 300 | SPX0_061 | PDX | 79,121,310 | 69,429,479 | 59,213,004 | 59,207,194 | 99.990 |
| 301 | SPX0_071 | PDX | 55,031,470 | 47,784,058 | 38,933,212 | 38,929,176 | 99.990 |
| 302 | SPX0_073 | PDX | 54,765,616 | 47,586,427 | 40,832,284 | 40,828,034 | 99.990 |
| 303 | SPX0_079 | PDX | 70,972,976 | 62,135,270 | 49,677,632 | 49,673,267 | 99.991 |
| 304 | SPX0_082 | PDX | 77,663,424 | 67,859,979 | 58,242,660 | 58,236,004 | 99.989 |
| 305 | SPX0_091 | PDX | 69,010,782 | 60,286,988 | 51,488,302 | 51,481,021 | 99.986 |
| 306 | SPX0_095 | PDX | 80,082,844 | 70,019,123 | 60,210,294 | 60,203,644 | 99.989 |
| 307 | SPX0_100 | PDX | 70,204,948 | 61,559,031 | 50,995,504 | 50,991,281 | 99.992 |
| 308 | SPX0_115 | PDX | 72,632,442 | 63,676,246 | 52,178,048 | 52,173,312 | 99.991 |
| 309 | SPX0_163 | PDX | 190,832,846 | 165,270,800 | 136,630,634 | 136,605,576 | 99.982 |
| 310 | SPX0_173 | PDX | 147,092,048 | 127,415,704 | 107,925,410 | 107,909,381 | 99.985 |
| 311 | SPX1_182 | PDX | 153,951,416 | 134,481,673 | 114,342,474 | 114,332,943 | 99.992 |
| 312 | SPX1_184 | PDX | 162,337,650 | 141,569,772 | 120,145,492 | 120,134,356 | 99.991 |
| 313 | SPX0_185 | PDX | 139,850,944 | 121,176,881 | 101,328,932 | 101,312,856 | 99.984 |
| 314 | SPX0_186 | PDX | 135,154,338 | 117,094,364 | 96,223,630 | 96,211,226 | 99.987 |
| 315 | SPX0_190 | PDX | 128,570,472 | 111,238,722 | 92,640,636 | 92,618,774 | 99.976 |
| 316 | SPX0_191 | PDX | 106,805,746 | 92,289,363 | 73,260,856 | 73,243,334 | 99.976 |
| 317 | SPX0_193 | PDX | 115,513,044 | 99,702,722 | 79,739,542 | 79,722,431 | 99.979 |
| 318 | SPX0_197 | PDX | 110,466,302 | 95,501,301 | 80,192,250 | 80,170,619 | 99.973 |
| 319 | SPX0_198 | PDX | 112,081,814 | 96,953,275 | 76,312,956 | 76,294,390 | 99.976 |
| 320 | SPX0_201 | PDX | 123,852,968 | 108,156,934 | 88,640,328 | 88,631,348 | 99.990 |
| 321 | SPX1_203 | PDX | 150,795,836 | 131,812,268 | 104,459,564 | 104,449,392 | 99.990 |
| 322 | SPX0_205 | PDX | 154,785,824 | 135,148,935 | 112,959,074 | 112,949,192 | 99.991 |
| 323 | SPX1_206 | PDX | 151,012,216 | 131,579,226 | 111,523,670 | 111,510,383 | 99.988 |
| 324 | SPX1_207 | PDX | 174,883,190 | 152,772,856 | 129,336,880 | 129,326,566 | 99.992 |
| 325 | SPX1_208 | PDX | 158,478,906 | 138,617,296 | 116,037,360 | 116,029,591 | 99.993 |
| 326 | SPX0_211 | PDX | 130,242,130 | 114,025,656 | 92,898,850 | 92,888,063 | 99.988 |
| 327 | SPX0_212 | PDX | 115,110,826 | 99,785,946 | 84,515,802 | 84,498,218 | 99.979 |
| 328 | SPX1_213 | PDX | 152,916,216 | 133,820,999 | 111,053,994 | 111,041,744 | 99.989 |
| 329 | SPX0_214 | PDX | 143,522,404 | 124,439,772 | 91,374,816 | 91,355,189 | 99.979 |
| 330 | SPX2_215 | PDX | 150,671,050 | 131,251,939 | 110,330,236 | 110,320,904 | 99.992 |
| 331 | SPX1_216 | PDX | 153,839,984 | 134,725,103 | 116,643,556 | 116,634,825 | 99.993 |
| 332 | SPX0_217 | PDX | 156,985,692 | 137,001,311 | 113,775,786 | 113,766,273 | 99.992 |
| 333 | SPX1_218 | PDX | 153,462,150 | 134,372,449 | 114,937,680 | 114,928,871 | 99.992 |
| 334 | SPX0_220 | PDX | 143,556,320 | 125,606,967 | 106,299,624 | 106,291,355 | 99.992 |
| 335 | SPX1_222 | PDX | 156,022,782 | 136,288,488 | 114,542,006 | 114,532,251 | 99.991 |
| 336 | SPX1_223 | PDX | 156,972,938 | 137,643,723 | 118,181,528 | 118,171,135 | 99.991 |
| 337 | SPX0_231 | PDX | 123,188,174 | 106,460,135 | 85,826,220 | 85,805,367 | 99.976 |
| 338 | SPX0_235 | PDX | 116,882,956 | 100,689,818 | 85,346,730 | 85,324,404 | 99.974 |
| 339 | SPX1_238 | PDX | 161,014,094 | 140,833,590 | 122,130,820 | 122,117,324 | 99.989 |
| 340 | SPX0_241 | PDX | 170,302,350 | 149,046,063 | 102,808,836 | 102,796,399 | 99.988 |
| 341 | SPX0_244 | PDX | 154,402,980 | 134,364,216 | 107,613,730 | 107,604,820 | 99.992 |
| 342 | SPX0_252 | PDX | 143,825,266 | 125,652,633 | 105,195,408 | 105,187,624 | 99.993 |
| 343 | SPX0_260 | PDX | 108,826,932 | 93,742,448 | 78,028,226 | 78,008,758 | 99.975 |
| 344 | SPX0_262 | PDX | 168,392,636 | 147,302,758 | 64,091,210 | 64,080,386 | 99.983 |
| 345 | SPX0_265 | PDX | 133,282,470 | 115,942,256 | 97,178,674 | 97,171,488 | 99.993 |
| 346 | SPX0_269 | PDX | 176,134,934 | 153,892,591 | 133,442,908 | 133,432,724 | 99.992 |
| 347 | SPX1_271 | PDX | 173,327,868 | 151,634,374 | 124,865,106 | 124,857,002 | 99.994 |
| 348 | SPX0_276 | PDX | 136,252,914 | 118,399,220 | 97,945,966 | 97,937,589 | 99.991 |
| 349 | SPX1_278 | PDX | 144,503,270 | 126,179,226 | 101,264,152 | 101,256,839 | 99.993 |
| 350 | SPX0_281 | PDX | 118,379,318 | 102,623,762 | 87,216,154 | 87,197,288 | 99.978 |
| 351 | SPX0_284 | PDX | 185,716,000 | 162,566,283 | 137,598,560 | 137,585,173 | 99.990 |
| 352 | SPX0_285 | PDX | 104,577,146 | 89,986,428 | 74,167,656 | 74,147,803 | 99.973 |
| 353 | SPX0_289 | PDX | 136,351,246 | 119,297,815 | 83,428,896 | 83,420,539 | 99.990 |
| 354 | SPX0_290 | PDX | 187,864,616 | 163,905,651 | 117,624,242 | 117,613,654 | 99.991 |
| 355 | SPX1_292 | PDX | 162,450,054 | 141,900,276 | 123,249,142 | 123,241,339 | 99.994 |
| 356 | SPX0_293 | PDX | 158,681,640 | 138,946,797 | 112,812,902 | 112,805,123 | 99.993 |
| 357 | SPX0_295 | PDX | 118,678,720 | 103,940,445 | 74,354,882 | 74,341,537 | 99.982 |
| 358 | SPX0_297 | PDX | 154,658,486 | 134,428,486 | 105,061,110 | 105,051,538 | 99.991 |
| 359 | SPX0_299 | PDX | 135,616,546 | 118,849,940 | 99,644,228 | 99,632,626 | 99.988 |
| 360 | SPX1_302 | PDX | 158,586,428 | 138,703,931 | 116,019,582 | 116,009,948 | 99.992 |
| 361 | SPX0_307 | PDX | 126,263,252 | 110,219,636 | 82,635,112 | 82,628,428 | 99.992 |
| 362 | SPX0_310 | PDX | 173,613,522 | 151,484,824 | 126,429,092 | 126,420,575 | 99.993 |
| 363 | SPX1_318 | PDX | 156,553,758 | 136,844,606 | 118,105,358 | 118,097,621 | 99.993 |
| 364 | SPX0_341 | PDX | 107,054,338 | 93,701,483 | 77,311,174 | 77,288,355 | 99.970 |
| 365 | SPX0_347 | PDX | 162,177,764 | 141,386,714 | 118,888,544 | 118,879,965 | 99.993 |
| 366 | SPX0_353 | PDX | 115,119,278 | 100,681,527 | 78,373,390 | 78,353,227 | 99.974 |
| 367 | SPX0_363 | PDX | 150,155,748 | 130,958,925 | 111,771,316 | 111,762,312 | 99.992 |
| 368 | SPX0_364 | PDX | 152,985,784 | 132,854,254 | 107,792,726 | 107,783,223 | 99.991 |
| 369 | SPX0_365 | PDX | 107,640,102 | 93,568,036 | 73,797,704 | 73,721,572 | 99.897 |
| 370 | SPX0_366 | PDX | 128,258,666 | 111,401,345 | 92,298,334 | 92,213,304 | 99.908 |
| 371 | SPX1_367 | PDX | 153,759,028 | 134,431,727 | 112,877,146 | 112,868,004 | 99.992 |
| 372 | SPX0_369 | PDX | 115,116,030 | 100,065,395 | 76,536,456 | 76,461,553 | 99.902 |
| 373 | SPX0_372 | PDX | 119,387,586 | 103,833,611 | 86,478,174 | 86,395,319 | 99.904 |
| 374 | SPX0_377 | PDX | 130,898,106 | 113,731,809 | 95,342,302 | 95,318,009 | 99.975 |
| 375 | SPX0_383 | PDX | 126,138,806 | 109,599,375 | 94,382,872 | 94,367,275 | 99.983 |
| 376 | SPX0_402 | PDX | 118,830,566 | 103,316,275 | 87,717,224 | 87,690,602 | 99.970 |
| 377 | SPX0_409 | PDX | 121,468,300 | 105,724,508 | 87,148,460 | 87,120,608 | 99.968 |
| 378 | SPX0_410 | PDX | 124,192,424 | 107,851,658 | 88,817,378 | 88,787,834 | 99.967 |
| 379 | SPX0_415 | PDX | 140,058,756 | 121,357,850 | 99,696,224 | 99,525,346 | 99.829 |
| 380 | SPX0_416 | PDX | 113,827,926 | 98,676,746 | 71,190,436 | 71,160,122 | 99.957 |
| 381 | SPX0_417 | PDX | 129,532,170 | 112,156,620 | 94,210,540 | 94,181,800 | 99.969 |
| 382 | SPX0_418 | PDX | 116,497,440 | 101,039,783 | 82,600,210 | 81,646,731 | 98.846 |
| 383 | SPX0_419 | PDX | 112,432,756 | 97,101,336 | 81,817,930 | 81,786,181 | 99.961 |
| 384 | SPX0_420 | PDX | 120,514,268 | 104,863,835 | 82,712,266 | 82,703,047 | 99.989 |
| 385 | SPX0_428 | PDX | 134,092,388 | 116,858,284 | 100,520,138 | 100,505,253 | 99.985 |
| 386 | SPX0_434 | PDX | 141,586,342 | 123,043,481 | 104,015,592 | 104,001,940 | 99.987 |
| 387 | SPX0_435 | PDX | 131,681,480 | 114,665,671 | 96,668,562 | 96,656,317 | 99.987 |
| 388 | SPX0_436 | PDX | 132,789,158 | 115,819,148 | 96,268,002 | 96,254,922 | 99.986 |
| 389 | SPX0_441 | PDX | 142,264,424 | 124,040,023 | 106,058,016 | 106,043,584 | 99.986 |
| 390 | SPX0_451 | PDX | 123,794,880 | 107,867,447 | 88,385,664 | 88,373,010 | 99.986 |
| 391 | SPX0_454 | PDX | 133,918,348 | 116,729,631 | 93,747,942 | 93,736,098 | 99.987 |
| 392 | SPX0_464 | PDX | 132,062,266 | 114,531,366 | 94,028,138 | 94,011,822 | 99.983 |
| 393 | SPX0_467 | PDX | 119,612,142 | 104,281,325 | 80,751,902 | 80,740,467 | 99.986 |
| 394 | SPX0_473 | PDX | 127,551,558 | 111,415,612 | 88,272,962 | 88,261,216 | 99.987 |
| 395 | SPX0_480 | PDX | 130,581,910 | 114,063,751 | 88,359,524 | 88,347,338 | 99.986 |
| 396 | SPX0_482 | PDX | 138,497,230 | 120,184,333 | 100,386,006 | 100,373,631 | 99.988 |
| 397 | DPX1_014 | PDX | 146,363,770 | 128,154,414 | 108,577,246 | 103,581,859 | 95.399 |
| 398 | DPX1_040 | PDX | 170,330,650 | 129,595,050 | 82,057,114 | 82,050,857 | 99.992 |
| 399 | DPX1_041 | PDX | 121,690,796 | 106,037,911 | 89,401,502 | 89,381,732 | 99.978 |
| 400 | DPX1_075 | PDX | 132,626,700 | 115,359,113 | 97,390,652 | 97,368,693 | 99.977 |
| 401 | SPX1_012 | PDX | 120,938,494 | 105,636,543 | 87,384,940 | 87,369,858 | 99.983 |
| 402 | SPX1_041 | PDX | 180,491,594 | 157,483,412 | 134,445,760 | 134,429,792 | 99.988 |
| 403 | SPX1_056 | PDX | 154,758,656 | 134,789,901 | 109,724,092 | 109,706,306 | 99.984 |
| 404 | SPX1_060 | PDX | 147,984,244 | 129,017,563 | 104,385,556 | 104,369,775 | 99.985 |
| 405 | SPX1_079 | PDX | 142,585,696 | 124,348,400 | 106,493,938 | 106,478,690 | 99.986 |
| 406 | SPX1_091 | PDX | 143,194,088 | 124,999,263 | 104,843,758 | 104,825,722 | 99.983 |
| 407 | SPX1_095 | PDX | 141,955,210 | 123,921,049 | 106,048,042 | 106,029,647 | 99.983 |
| 408 | SPX1_100 | PDX | 148,769,620 | 129,866,157 | 108,224,374 | 108,210,939 | 99.988 |
| 409 | SPX1_163 | PDX | 167,611,490 | 146,393,482 | 102,014,750 | 102,005,683 | 99.991 |
| 410 | SPX1_173 | PDX | 164,750,354 | 143,851,102 | 123,376,968 | 123,367,724 | 99.993 |
| 411 | SPX1_185 | PDX | 177,916,150 | 155,887,010 | 123,726,096 | 109,564,293 | 88.554 |
| 412 | SPX1_186 | PDX | 156,731,612 | 119,767,963 | 87,762,412 | 87,757,751 | 99.995 |
| 413 | SPX1_190 | PDX | 149,601,150 | 131,277,526 | 98,495,126 | 98,486,427 | 99.991 |
| 414 | SPX1_193 | PDX | 150,253,520 | 131,193,428 | 108,692,590 | 108,687,467 | 99.995 |
| 415 | SPX1_197 | PDX | 147,941,942 | 128,550,085 | 110,400,060 | 110,389,799 | 99.991 |
| 416 | SPX1_214 | PDX | 150,470,294 | 131,571,896 | 106,346,092 | 106,337,816 | 99.992 |
| 417 | SPX1_231 | PDX | 160,709,494 | 140,390,663 | 121,753,344 | 121,744,622 | 99.993 |
| 418 | SPX1_276 | PDX | 138,106,440 | 120,902,540 | 104,096,802 | 104,087,560 | 99.991 |
| 419 | SPX1_297 | PDX | 142,266,262 | 123,890,393 | 103,710,374 | 103,699,366 | 99.989 |
| 420 | SPX1_299 | PDX | 136,592,436 | 119,634,590 | 100,153,212 | 100,146,675 | 99.993 |
| 421 | SPX1_310 | PDX | 217,012,628 | 196,050,752 | 168,077,242 | 167,977,533 | 99.941 |
| 422 | SPX1_353 | PDX | 150,725,586 | 131,808,976 | 107,690,116 | 107,684,047 | 99.994 |
| 423 | SPX1_369 | PDX | 164,865,564 | 144,170,779 | 121,737,710 | 121,729,947 | 99.994 |
| 424 | SPX1_416 | PDX | 149,451,518 | 129,870,322 | 111,171,316 | 111,155,576 | 99.986 |
| 425 | SPX2_012 | PDX | 166,696,630 | 144,869,652 | 120,999,218 | 120,985,157 | 99.988 |
| 426 | SPX2_041 | PDX | 156,003,530 | 135,594,361 | 110,900,116 | 110,884,993 | 99.986 |
| 427 | SPX2_056 | PDX | 170,531,596 | 148,465,394 | 125,607,322 | 125,590,501 | 99.987 |
| 428 | SPX2_060 | PDX | 153,050,134 | 133,093,167 | 110,965,806 | 110,950,815 | 99.986 |
| 429 | SPX2_079 | PDX | 161,081,094 | 139,826,270 | 113,123,776 | 113,112,507 | 99.990 |
| 430 | SPX2_091 | PDX | 151,122,834 | 131,167,197 | 110,388,350 | 110,380,234 | 99.993 |
| 431 | SPX2_095 | PDX | 154,634,282 | 134,121,262 | 114,777,290 | 114,758,909 | 99.984 |
| 432 | SPX2_100 | PDX | 144,652,070 | 126,651,006 | 106,057,852 | 106,052,028 | 99.995 |
| 433 | SPX3_012 | PDX | 148,852,802 | 129,407,466 | 109,216,322 | 109,200,701 | 99.986 |
| 434 | SPX3_041 | PDX | 150,494,330 | 131,225,183 | 111,923,548 | 111,912,670 | 99.990 |
| 435 | SPX3_056 | PDX | 168,894,504 | 147,509,213 | 100,842,048 | 100,825,373 | 99.983 |
| 436 | SPX3_060 | PDX | 147,559,176 | 128,600,832 | 107,435,108 | 107,420,280 | 99.986 |
| 437 | SPX3_079 | PDX | 151,794,836 | 132,296,807 | 110,747,148 | 110,734,353 | 99.988 |
| 438 | SPX3_091 | PDX | 154,653,326 | 134,588,901 | 109,399,190 | 109,383,475 | 99.986 |
| 439 | SPX3_095 | PDX | 156,117,120 | 136,735,151 | 116,057,708 | 116,049,092 | 99.993 |
| 440 | SPX3_100 | PDX | 159,139,908 | 139,290,442 | 117,225,654 | 117,218,782 | 99.994 |

PDX, patient-derived xenograft.

**Supplementary Table 2.** Result of Xenome alignment to human and mouse references

|  | **Sample** | **Human** | **%** | **Mouse** | **%** | **Both** | **%** | **Neither** | **%** | **Ambiguous** | **%** |
| --- | --- | --- | --- | --- | --- | --- | --- | --- | --- | --- | --- |
| 1 | SPX0_012 | 24,078,154 | 96.74 | 637,141 | 2.56 | 6,954 | 0.028 | 21,302 | 0.086 | 147,105 | 0.591 |
| 2 | SPX0_013 | 23,603,869 | 96.29 | 725,832 | 2.96 | 8,003 | 0.033 | 22,568 | 0.092 | 154,145 | 0.629 |
| 3 | SPX0_029 | 19,059,659 | 95.15 | 830,605 | 4.15 | 6,331 | 0.032 | 15,953 | 0.080 | 119,383 | 0.596 |
| 4 | SPX0_041 | 23,352,043 | 97.10 | 534,970 | 2.22 | 7,214 | 0.030 | 20,310 | 0.084 | 134,764 | 0.560 |
| 5 | SPX0_044 | 23,034,168 | 97.91 | 333,256 | 1.42 | 6,588 | 0.028 | 21,848 | 0.093 | 131,067 | 0.557 |
| 6 | SPX0_056 | 22,184,622 | 94.03 | 1,210,001 | 5.13 | 7,902 | 0.033 | 20,588 | 0.087 | 170,495 | 0.723 |
| 7 | SPX0_071 | 19,466,606 | 93.77 | 1,134,914 | 5.47 | 7,063 | 0.034 | 15,206 | 0.073 | 136,950 | 0.660 |
| 8 | SPX0_073 | 20,416,142 | 98.70 | 145,843 | 0.71 | 5,046 | 0.024 | 14,262 | 0.069 | 104,611 | 0.506 |
| 9 | SPX0_008 | 37,642,470 | 93.61 | 2,278,468 | 5.67 | 14,968 | 0.037 | 36,137 | 0.090 | 241,626 | 0.601 |
| 10 | SPX0_055 | 21,742,270 | 76.08 | 6,573,381 | 23.00 | 20,746 | 0.073 | 19,575 | 0.068 | 223,068 | 0.781 |
| 11 | SPX0_060 | 24,791,353 | 82.78 | 4,893,808 | 16.34 | 18,254 | 0.061 | 24,676 | 0.082 | 220,212 | 0.735 |
| 12 | SPX0_061 | 29,606,502 | 97.13 | 670,370 | 2.20 | 9,701 | 0.032 | 26,361 | 0.086 | 169,221 | 0.555 |
| 13 | SPX0_079 | 24,838,816 | 91.26 | 2,175,715 | 7.99 | 13,114 | 0.048 | 22,940 | 0.084 | 167,559 | 0.616 |
| 14 | SPX0_095 | 30,105,147 | 98.27 | 305,088 | 1.00 | 13,836 | 0.045 | 40,003 | 0.131 | 171,259 | 0.559 |
| 15 | SPX0_100 | 25,497,752 | 94.41 | 1,326,525 | 4.91 | 9,741 | 0.036 | 18,674 | 0.069 | 154,243 | 0.571 |
| 16 | SPX0_115 | 26,089,024 | 93.40 | 1,646,497 | 5.89 | 11,746 | 0.042 | 21,847 | 0.078 | 163,792 | 0.586 |
| 17 | SPX0_023 | 31,269,089 | 93.98 | 1,697,644 | 5.10 | 11,306 | 0.034 | 35,364 | 0.106 | 257,925 | 0.775 |
| 18 | SPX0_082 | 29,121,330 | 98.14 | 358,031 | 1.21 | 9,344 | 0.031 | 25,204 | 0.085 | 157,865 | 0.532 |
| 19 | SPX0_091 | 25,744,151 | 97.69 | 416,767 | 1.58 | 8,901 | 0.034 | 32,018 | 0.122 | 150,044 | 0.569 |
| 20 | SPX0_190 | 46,320,318 | 96.09 | 1,455,004 | 3.02 | 14,997 | 0.031 | 54,194 | 0.112 | 359,118 | 0.745 |
| 21 | SPX0_191 | 36,630,428 | 91.71 | 2,833,122 | 7.09 | 15,292 | 0.038 | 40,589 | 0.102 | 423,024 | 1.059 |
| 22 | SPX0_193 | 39,869,771 | 92.49 | 2,771,367 | 6.43 | 14,585 | 0.034 | 33,626 | 0.078 | 419,155 | 0.972 |
| 23 | SPX0_197 | 40,096,125 | 96.95 | 881,968 | 2.13 | 11,324 | 0.027 | 42,936 | 0.104 | 325,217 | 0.786 |
| 24 | SPX0_198 | 38,156,478 | 90.84 | 3,309,447 | 7.88 | 16,562 | 0.039 | 53,511 | 0.127 | 469,175 | 1.117 |
| 25 | SPX0_212 | 42,257,901 | 97.56 | 687,532 | 1.59 | 12,619 | 0.029 | 34,443 | 0.080 | 322,309 | 0.744 |
| 26 | SPX0_214 | 45,687,408 | 84.55 | 7,499,616 | 13.88 | 25,885 | 0.048 | 38,448 | 0.071 | 781,939 | 1.447 |
| 27 | SPX0_231 | 42,913,110 | 93.15 | 2,644,273 | 5.74 | 17,173 | 0.037 | 51,175 | 0.111 | 445,174 | 0.966 |
| 28 | SPX0_235 | 42,673,365 | 98.20 | 422,314 | 0.97 | 13,085 | 0.030 | 46,074 | 0.106 | 300,330 | 0.691 |
| 29 | SPX0_260 | 39,014,113 | 96.45 | 1,033,181 | 2.55 | 12,272 | 0.030 | 39,224 | 0.097 | 352,406 | 0.871 |
| 30 | SPX0_285 | 37,083,828 | 95.60 | 1,304,442 | 3.36 | 12,490 | 0.032 | 37,470 | 0.097 | 353,201 | 0.911 |
| 31 | SPX0_163 | 68,315,317 | 95.36 | 2,625,398 | 3.66 | 25,094 | 0.035 | 98,693 | 0.138 | 574,265 | 0.802 |
| 32 | SPX0_173 | 53,962,705 | 97.68 | 869,150 | 1.57 | 15,705 | 0.028 | 50,956 | 0.092 | 346,992 | 0.628 |
| 33 | SPX0_185 | 50,664,466 | 96.40 | 1,439,528 | 2.74 | 17,022 | 0.032 | 58,507 | 0.111 | 375,589 | 0.715 |
| 34 | SPX0_186 | 48,111,815 | 94.75 | 2,184,474 | 4.30 | 15,597 | 0.031 | 38,851 | 0.077 | 427,022 | 0.841 |
| 35 | SPX1_012 | 43,692,470 | 94.67 | 1,970,142 | 4.27 | 15,121 | 0.033 | 46,868 | 0.102 | 426,086 | 0.923 |
| 36 | SPX1_079 | 53,246,969 | 98.14 | 597,204 | 1.10 | 22,217 | 0.041 | 49,060 | 0.090 | 340,205 | 0.627 |
| 37 | SPX1_091 | 52,421,879 | 96.03 | 1,672,821 | 3.06 | 17,825 | 0.033 | 63,916 | 0.117 | 411,254 | 0.753 |
| 38 | SPX1_095 | 53,024,021 | 97.97 | 676,866 | 1.25 | 18,590 | 0.034 | 58,898 | 0.109 | 346,463 | 0.640 |
| 39 | SPX1_100 | 54,112,187 | 95.42 | 2,087,891 | 3.68 | 17,098 | 0.030 | 37,657 | 0.066 | 457,591 | 0.807 |
| 40 | SPX1_041 | 67,222,880 | 97.73 | 1,007,843 | 1.47 | 22,784 | 0.033 | 82,502 | 0.120 | 451,656 | 0.657 |
| 41 | SPX1_056 | 54,862,046 | 93.35 | 3,230,475 | 5.50 | 23,118 | 0.039 | 80,235 | 0.137 | 574,800 | 0.978 |
| 42 | SPX1_060 | 52,192,778 | 92.68 | 3,452,697 | 6.13 | 22,677 | 0.040 | 67,249 | 0.119 | 576,806 | 1.024 |
| 43 | SPX2_012 | 60,499,609 | 96.00 | 2,027,860 | 3.22 | 19,778 | 0.031 | 52,833 | 0.084 | 420,356 | 0.667 |
| 44 | SPX2_041 | 55,450,058 | 94.01 | 2,907,047 | 4.93 | 20,430 | 0.035 | 51,442 | 0.087 | 554,608 | 0.940 |
| 45 | SPX2_056 | 62,803,661 | 97.08 | 1,378,817 | 2.13 | 19,307 | 0.030 | 62,419 | 0.096 | 426,644 | 0.660 |
| 46 | SPX2_060 | 55,482,903 | 95.77 | 1,945,610 | 3.36 | 18,868 | 0.033 | 46,153 | 0.080 | 440,462 | 0.760 |
| 47 | SPX2_079 | 56,561,888 | 93.07 | 3,674,301 | 6.05 | 24,331 | 0.040 | 45,513 | 0.075 | 465,985 | 0.767 |
| 48 | SPX2_091 | 55,194,175 | 96.86 | 1,427,125 | 2.50 | 23,306 | 0.041 | 64,740 | 0.114 | 273,849 | 0.481 |
| 49 | SPX2_095 | 57,388,645 | 98.51 | 425,785 | 0.73 | 20,324 | 0.035 | 59,125 | 0.101 | 360,281 | 0.618 |
| 50 | SPX2_100 | 53,028,926 | 95.38 | 2,184,640 | 3.93 | 33,641 | 0.061 | 39,553 | 0.071 | 310,899 | 0.559 |
| 51 | SPX3_012 | 54,608,161 | 96.98 | 1,224,238 | 2.17 | 17,305 | 0.031 | 52,359 | 0.093 | 405,600 | 0.720 |
| 52 | SPX3_041 | 55,961,774 | 97.71 | 909,039 | 1.59 | 19,737 | 0.034 | 47,052 | 0.082 | 333,941 | 0.583 |
| 53 | SPX3_056 | 50,421,024 | 78.18 | 12,937,876 | 20.06 | 41,013 | 0.064 | 64,400 | 0.100 | 1,026,490 | 1.592 |
| 54 | SPX3_060 | 53,717,554 | 95.75 | 1,864,864 | 3.32 | 22,070 | 0.039 | 53,388 | 0.095 | 444,278 | 0.792 |
| 55 | SPX3_079 | 55,373,574 | 95.95 | 1,825,790 | 3.16 | 21,471 | 0.037 | 45,012 | 0.078 | 443,052 | 0.768 |
| 56 | SPX3_091 | 54,699,595 | 93.19 | 3,395,431 | 5.78 | 22,919 | 0.039 | 67,668 | 0.115 | 512,597 | 0.873 |
| 57 | SPX3_095 | 58,028,854 | 96.63 | 1,581,507 | 2.63 | 37,421 | 0.062 | 66,128 | 0.110 | 337,547 | 0.562 |
| 58 | SPX3_100 | 58,612,827 | 95.89 | 2,102,382 | 3.44 | 34,594 | 0.057 | 46,833 | 0.077 | 327,282 | 0.535 |
| 59 | SPX1_163 | 51,007,375 | 79.70 | 12,319,981 | 19.25 | 61,355 | 0.096 | 64,704 | 0.101 | 549,850 | 0.859 |
| 60 | SPX1_173 | 61,688,484 | 98.10 | 783,828 | 1.25 | 25,176 | 0.040 | 47,658 | 0.076 | 335,815 | 0.534 |
| 61 | SPX1_182 | 57,171,237 | 97.24 | 1,230,782 | 2.09 | 27,541 | 0.047 | 59,258 | 0.101 | 304,093 | 0.517 |
| 62 | SPX1_184 | 60,072,746 | 97.24 | 1,300,115 | 2.10 | 19,249 | 0.031 | 51,665 | 0.084 | 331,815 | 0.537 |
| 63 | SPX1_197 | 55,200,030 | 98.77 | 325,526 | 0.58 | 23,252 | 0.042 | 62,042 | 0.111 | 276,700 | 0.495 |
| 64 | SPX1_206 | 55,761,835 | 97.21 | 1,204,683 | 2.10 | 19,706 | 0.034 | 56,449 | 0.098 | 316,826 | 0.552 |
| 65 | DPX1_014 | 54,288,623 | 96.59 | 1,574,812 | 2.80 | 25,894 | 0.046 | 49,846 | 0.089 | 268,394 | 0.478 |
| 66 | DPX1_029 | 53,344,583 | 88.89 | 6,196,668 | 10.33 | 37,274 | 0.062 | 52,585 | 0.088 | 381,980 | 0.636 |
| 67 | DPX1_040 | 41,028,557 | 85.26 | 6,686,778 | 13.90 | 38,201 | 0.079 | 47,852 | 0.099 | 318,590 | 0.662 |
| 68 | SPX1_185 | 61,863,048 | 90.37 | 6,071,288 | 8.87 | 45,564 | 0.067 | 69,875 | 0.102 | 404,439 | 0.591 |
| 69 | SPX1_186 | 43,881,206 | 97.71 | 779,069 | 1.73 | 19,212 | 0.043 | 37,759 | 0.084 | 192,347 | 0.428 |
| 70 | SPX1_190 | 49,247,563 | 85.36 | 7,882,594 | 13.66 | 43,238 | 0.075 | 181,252 | 0.314 | 338,165 | 0.586 |
| 71 | SPX1_193 | 54,346,295 | 94.70 | 2,688,670 | 4.68 | 32,657 | 0.057 | 38,516 | 0.067 | 283,929 | 0.495 |
| 72 | SPX1_203 | 52,229,782 | 90.50 | 4,943,606 | 8.57 | 35,301 | 0.061 | 62,853 | 0.109 | 440,240 | 0.763 |
| 73 | SPX1_207 | 64,668,440 | 96.77 | 1,701,834 | 2.55 | 30,612 | 0.046 | 55,895 | 0.084 | 366,975 | 0.549 |
| 74 | SPX1_208 | 58,018,680 | 95.55 | 2,320,910 | 3.82 | 32,053 | 0.053 | 64,874 | 0.107 | 285,217 | 0.470 |
| 75 | SPX1_212 | 9,928,470 | 76.19 | 2,980,569 | 22.87 | 12,304 | 0.094 | 10,605 | 0.081 | 99,079 | 0.760 |
| 76 | SPX1_213 | 55,526,997 | 94.76 | 2,650,564 | 4.52 | 23,254 | 0.040 | 62,834 | 0.107 | 335,269 | 0.572 |
| 77 | SPX1_214 | 53,173,046 | 92.32 | 3,998,185 | 6.94 | 33,026 | 0.057 | 67,965 | 0.118 | 323,859 | 0.562 |
| 78 | SPX1_216 | 58,321,778 | 98.75 | 409,028 | 0.69 | 27,159 | 0.046 | 54,473 | 0.092 | 250,437 | 0.424 |
| 79 | SPX1_218 | 57,468,840 | 97.57 | 1,089,701 | 1.85 | 25,628 | 0.044 | 64,961 | 0.110 | 251,972 | 0.428 |
| 80 | SPX1_223 | 59,090,764 | 97.83 | 946,059 | 1.57 | 19,879 | 0.033 | 45,314 | 0.075 | 296,657 | 0.491 |
| 81 | SPX1_231 | 60,876,672 | 99.17 | 163,629 | 0.27 | 25,980 | 0.042 | 62,647 | 0.102 | 255,939 | 0.417 |
| 82 | SPX1_238 | 61,065,410 | 99.02 | 247,090 | 0.40 | 24,149 | 0.039 | 64,962 | 0.105 | 268,561 | 0.435 |
| 83 | SPX1_271 | 62,432,553 | 94.03 | 3,512,819 | 5.29 | 34,347 | 0.052 | 50,801 | 0.077 | 364,250 | 0.549 |
| 84 | SPX1_276 | 52,048,401 | 98.20 | 621,722 | 1.17 | 24,492 | 0.046 | 51,891 | 0.098 | 256,428 | 0.484 |
| 85 | SPX1_278 | 50,632,076 | 91.82 | 4,116,118 | 7.46 | 32,292 | 0.059 | 60,187 | 0.109 | 302,488 | 0.549 |
| 86 | SPX1_292 | 61,624,571 | 99.29 | 99,054 | 0.16 | 27,290 | 0.044 | 56,636 | 0.091 | 256,887 | 0.414 |
| 87 | SPX1_299 | 50,076,606 | 95.42 | 2,075,478 | 3.95 | 28,116 | 0.054 | 45,946 | 0.088 | 255,189 | 0.486 |
| 88 | SPX1_318 | 59,052,679 | 98.61 | 485,151 | 0.81 | 26,779 | 0.045 | 58,999 | 0.099 | 260,165 | 0.434 |
| 89 | SPX1_353 | 53,845,058 | 93.29 | 3,489,017 | 6.05 | 32,682 | 0.057 | 39,838 | 0.069 | 309,118 | 0.536 |
| 90 | SPX1_367 | 56,438,573 | 95.90 | 2,018,057 | 3.43 | 30,953 | 0.053 | 70,829 | 0.120 | 294,880 | 0.501 |
| 91 | SPX1_369 | 60,868,855 | 96.43 | 1,850,180 | 2.93 | 29,907 | 0.047 | 47,122 | 0.075 | 328,857 | 0.521 |
| 92 | SPX1_310 | 84,038,621 | 93.96 | 4,079,909 | 4.56 | 46,326 | 0.052 | 181,403 | 0.203 | 1,092,304 | 1.221 |
| 93 | DPX0_005 | 58,790,506 | 97.54 | 1,087,870 | 1.80 | 28,874 | 0.048 | 68,323 | 0.113 | 296,414 | 0.492 |
| 94 | DPX0_056 | 72,054,578 | 98.91 | 346,991 | 0.48 | 30,225 | 0.041 | 72,405 | 0.099 | 345,221 | 0.474 |
| 95 | DPX0_057 | 70,134,139 | 98.79 | 422,518 | 0.60 | 31,222 | 0.044 | 73,864 | 0.104 | 333,934 | 0.470 |
| 96 | SPX0_241 | 51,404,418 | 78.68 | 12,933,747 | 19.80 | 65,494 | 0.100 | 83,281 | 0.127 | 844,080 | 1.292 |
| 97 | SPX0_262 | 32,045,605 | 49.65 | 31,427,314 | 48.69 | 139,856 | 0.217 | 72,752 | 0.113 | 862,118 | 1.336 |
| 98 | SPX0_284 | 68,799,280 | 96.56 | 1,898,580 | 2.66 | 36,743 | 0.052 | 76,172 | 0.107 | 438,997 | 0.616 |
| 99 | SPX0_289 | 41,714,448 | 79.80 | 9,875,756 | 18.89 | 52,786 | 0.101 | 55,233 | 0.106 | 577,481 | 1.105 |
| 100 | SPX0_293 | 56,406,451 | 92.60 | 4,052,171 | 6.65 | 40,410 | 0.066 | 63,367 | 0.104 | 350,132 | 0.575 |
| 101 | SPX0_307 | 41,317,556 | 85.75 | 6,405,131 | 13.29 | 43,979 | 0.091 | 53,963 | 0.112 | 365,824 | 0.759 |
| 102 | SPX0_310 | 63,214,546 | 95.54 | 2,474,192 | 3.74 | 37,340 | 0.056 | 76,519 | 0.116 | 365,436 | 0.552 |
| 103 | SPX0_347 | 59,444,272 | 96.31 | 1,847,726 | 2.99 | 32,319 | 0.052 | 71,419 | 0.116 | 328,574 | 0.532 |
| 104 | SPX0_363 | 55,885,658 | 97.70 | 932,365 | 1.63 | 31,615 | 0.055 | 64,411 | 0.113 | 289,764 | 0.507 |
| 105 | DPX0_024 | 47,415,446 | 88.43 | 5,648,343 | 10.53 | 30,859 | 0.058 | 39,989 | 0.075 | 484,580 | 0.904 |
| 106 | DPX1_041 | 44,700,751 | 96.75 | 1,170,379 | 2.53 | 19,085 | 0.041 | 54,691 | 0.118 | 259,631 | 0.562 |
| 107 | DPX1_075 | 48,695,326 | 97.05 | 1,136,556 | 2.27 | 21,799 | 0.043 | 50,339 | 0.100 | 269,421 | 0.537 |
| 108 | SPX1_297 | 51,855,187 | 96.13 | 1,730,741 | 3.21 | 23,177 | 0.043 | 29,644 | 0.055 | 302,357 | 0.561 |
| 109 | SPX1_416 | 55,585,658 | 98.52 | 478,154 | 0.85 | 25,824 | 0.046 | 51,158 | 0.091 | 278,675 | 0.494 |
| 110 | SPX0_201 | 44,320,164 | 93.70 | 2,665,682 | 5.64 | 19,221 | 0.041 | 21,840 | 0.046 | 272,440 | 0.576 |
| 111 | SPX0_211 | 46,449,425 | 92.88 | 3,217,641 | 6.43 | 21,063 | 0.042 | 33,388 | 0.067 | 288,955 | 0.578 |
| 112 | SPX0_281 | 43,608,077 | 97.82 | 587,804 | 1.32 | 15,285 | 0.034 | 74,900 | 0.168 | 294,962 | 0.662 |
| 113 | SPX0_295 | 37,177,441 | 81.54 | 7,990,518 | 17.53 | 30,997 | 0.068 | 37,852 | 0.083 | 357,229 | 0.783 |
| 114 | SPX0_299 | 49,822,114 | 95.52 | 1,988,993 | 3.81 | 18,255 | 0.035 | 39,704 | 0.076 | 290,243 | 0.556 |
| 115 | SPX0_341 | 38,655,587 | 94.10 | 2,133,843 | 5.19 | 16,122 | 0.039 | 31,591 | 0.077 | 242,173 | 0.590 |
| 116 | SPX0_353 | 39,186,695 | 88.86 | 4,559,873 | 10.34 | 21,468 | 0.049 | 27,316 | 0.062 | 303,054 | 0.687 |
| 117 | DPX0_014 | 39,193,408 | 84.42 | 6,833,004 | 14.72 | 29,008 | 0.062 | 38,239 | 0.082 | 332,920 | 0.717 |
| 118 | DPX0_031 | 42,101,932 | 96.83 | 1,110,479 | 2.55 | 16,535 | 0.038 | 27,708 | 0.064 | 224,623 | 0.517 |
| 119 | DPX0_032 | 39,976,817 | 89.81 | 4,193,082 | 9.42 | 20,945 | 0.047 | 34,155 | 0.077 | 289,260 | 0.650 |
| 120 | DPX0_039 | 43,398,158 | 92.47 | 3,199,162 | 6.82 | 20,870 | 0.044 | 25,731 | 0.055 | 289,147 | 0.616 |
| 121 | DPX0_040 | 40,676,856 | 88.66 | 4,814,945 | 10.50 | 26,952 | 0.059 | 44,407 | 0.097 | 314,756 | 0.686 |
| 122 | DPX0_041 | 37,891,949 | 80.24 | 8,871,046 | 18.79 | 35,193 | 0.075 | 45,835 | 0.097 | 379,706 | 0.804 |
| 123 | SPX0_365 | 36,898,852 | 90.70 | 3,435,693 | 8.44 | 20,377 | 0.050 | 40,088 | 0.099 | 288,299 | 0.709 |
| 124 | SPX0_366 | 46,149,167 | 95.37 | 1,897,217 | 3.92 | 17,347 | 0.036 | 38,289 | 0.079 | 288,152 | 0.595 |
| 125 | SPX0_369 | 38,268,228 | 87.95 | 4,878,246 | 11.21 | 24,252 | 0.056 | 28,844 | 0.066 | 311,664 | 0.716 |
| 126 | SPX0_372 | 43,239,087 | 95.71 | 1,614,568 | 3.57 | 19,266 | 0.043 | 38,761 | 0.086 | 263,687 | 0.584 |
| 127 | SPX0_377 | 47,671,151 | 96.43 | 1,425,485 | 2.88 | 19,257 | 0.039 | 38,724 | 0.078 | 279,678 | 0.566 |
| 128 | SPX0_402 | 43,858,612 | 97.59 | 788,457 | 1.75 | 16,002 | 0.036 | 41,369 | 0.092 | 238,427 | 0.531 |
| 129 | SPX0_409 | 43,574,230 | 94.64 | 2,154,704 | 4.68 | 18,615 | 0.040 | 29,291 | 0.064 | 263,399 | 0.572 |
| 130 | SPX0_410 | 44,408,689 | 94.77 | 2,110,425 | 4.50 | 18,377 | 0.039 | 40,896 | 0.087 | 281,606 | 0.601 |
| 131 | SPX0_416 | 35,595,218 | 83.18 | 6,780,036 | 15.84 | 30,627 | 0.072 | 45,653 | 0.107 | 344,003 | 0.804 |
| 132 | SPX0_417 | 47,105,270 | 96.96 | 1,129,478 | 2.32 | 17,739 | 0.037 | 44,381 | 0.091 | 283,960 | 0.585 |
| 133 | SPX0_418 | 41,300,105 | 94.19 | 2,211,149 | 5.04 | 18,412 | 0.042 | 53,777 | 0.123 | 265,760 | 0.606 |
| 134 | SPX0_419 | 40,908,965 | 97.50 | 708,841 | 1.69 | 16,588 | 0.040 | 62,880 | 0.150 | 259,086 | 0.618 |
| 135 | SPX0_420 | 41,356,133 | 90.60 | 3,961,306 | 8.68 | 23,227 | 0.051 | 28,185 | 0.062 | 275,940 | 0.605 |
| 136 | SPX0_428 | 50,260,069 | 98.66 | 357,365 | 0.70 | 15,756 | 0.031 | 45,963 | 0.090 | 263,382 | 0.517 |
| 137 | SPX0_434 | 52,007,796 | 97.22 | 1,123,105 | 2.10 | 21,057 | 0.039 | 57,765 | 0.108 | 282,597 | 0.528 |
| 138 | SPX0_435 | 48,334,281 | 96.77 | 1,296,186 | 2.60 | 16,947 | 0.034 | 39,794 | 0.080 | 260,859 | 0.522 |
| 139 | SPX0_436 | 48,134,001 | 95.27 | 2,050,652 | 4.06 | 17,341 | 0.034 | 34,355 | 0.068 | 288,678 | 0.571 |
| 140 | SPX0_441 | 53,029,008 | 98.01 | 709,569 | 1.31 | 18,412 | 0.034 | 66,171 | 0.122 | 283,201 | 0.523 |
| 141 | SPX0_451 | 44,192,832 | 93.98 | 2,489,884 | 5.29 | 21,629 | 0.046 | 39,737 | 0.085 | 280,858 | 0.597 |
| 142 | SPX0_454 | 46,873,971 | 92.08 | 3,669,090 | 7.21 | 23,770 | 0.047 | 41,830 | 0.082 | 295,396 | 0.580 |
| 143 | SPX0_467 | 40,375,951 | 88.79 | 4,722,635 | 10.39 | 23,816 | 0.052 | 36,937 | 0.081 | 315,098 | 0.693 |
| 144 | SPX0_473 | 44,136,481 | 90.66 | 4,179,745 | 8.59 | 26,017 | 0.053 | 41,741 | 0.086 | 300,923 | 0.618 |
| 145 | SPX0_480 | 44,179,762 | 88.63 | 5,293,732 | 10.62 | 27,764 | 0.056 | 39,834 | 0.080 | 307,063 | 0.616 |
| 146 | DPX0_044 | 51,551,801 | 94.49 | 2,588,030 | 4.74 | 24,676 | 0.045 | 48,822 | 0.089 | 344,124 | 0.631 |
| 147 | DPX0_048 | 45,985,184 | 92.79 | 3,190,044 | 6.44 | 23,121 | 0.047 | 49,837 | 0.101 | 310,791 | 0.627 |
| 148 | DPX0_060 | 49,054,771 | 94.22 | 2,646,652 | 5.08 | 22,624 | 0.043 | 30,203 | 0.058 | 308,461 | 0.592 |
| 149 | DPX0_075 | 46,081,926 | 94.56 | 2,314,322 | 4.75 | 17,574 | 0.036 | 40,939 | 0.084 | 277,312 | 0.569 |
| 150 | DPX0_078 | 40,017,978 | 79.16 | 10,069,077 | 19.92 | 40,902 | 0.081 | 40,478 | 0.080 | 387,110 | 0.766 |
| 151 | DPX0_081 | 46,845,649 | 90.55 | 4,520,512 | 8.74 | 23,891 | 0.046 | 39,320 | 0.076 | 302,909 | 0.586 |
| 152 | DPX0_084 | 49,700,891 | 94.86 | 2,334,373 | 4.46 | 21,179 | 0.040 | 41,633 | 0.079 | 296,911 | 0.567 |
| 153 | DPX0_085 | 49,859,091 | 92.57 | 3,633,632 | 6.75 | 21,994 | 0.041 | 37,165 | 0.069 | 311,626 | 0.579 |
| 154 | DPX0_088 | 54,461,314 | 96.13 | 1,810,823 | 3.20 | 19,796 | 0.035 | 42,306 | 0.075 | 320,213 | 0.565 |
| 155 | DPX0_089 | 52,773,765 | 97.90 | 769,761 | 1.43 | 20,095 | 0.037 | 55,387 | 0.103 | 284,750 | 0.528 |
| 156 | DPX0_091 | 43,708,070 | 93.41 | 2,758,249 | 5.89 | 20,418 | 0.044 | 32,947 | 0.070 | 274,024 | 0.586 |
| 157 | DPX0_094 | 43,587,766 | 85.65 | 6,863,728 | 13.49 | 29,545 | 0.058 | 42,173 | 0.083 | 366,488 | 0.720 |
| 158 | SPX0_220 | 53,149,812 | 96.56 | 1,523,252 | 2.77 | 30,802 | 0.056 | 56,527 | 0.103 | 283,262 | 0.515 |
| 159 | SPX0_252 | 52,597,704 | 95.71 | 1,968,012 | 3.58 | 28,863 | 0.053 | 49,593 | 0.090 | 310,887 | 0.566 |
| 160 | SPX0_269 | 66,721,454 | 99.11 | 181,490 | 0.27 | 33,271 | 0.049 | 78,173 | 0.116 | 307,868 | 0.457 |
| 161 | SPX0_244 | 53,806,865 | 91.92 | 4,275,618 | 7.30 | 20,400 | 0.035 | 34,210 | 0.058 | 398,660 | 0.681 |
| 162 | SPX0_265 | 48,589,337 | 96.22 | 1,580,933 | 3.13 | 16,951 | 0.034 | 30,599 | 0.061 | 282,493 | 0.559 |
| 163 | SPX0_276 | 48,972,983 | 95.08 | 2,166,207 | 4.21 | 17,202 | 0.033 | 33,576 | 0.065 | 317,797 | 0.617 |
| 164 | SPX0_297 | 52,530,555 | 89.80 | 5,456,611 | 9.33 | 25,053 | 0.043 | 38,433 | 0.066 | 447,370 | 0.765 |
| 165 | SPX0_364 | 53,896,363 | 93.31 | 3,430,412 | 5.94 | 21,305 | 0.037 | 36,281 | 0.063 | 374,309 | 0.648 |
| 166 | SPX0_383 | 47,191,436 | 98.93 | 203,657 | 0.43 | 14,784 | 0.031 | 39,313 | 0.082 | 254,478 | 0.533 |
| 167 | SPX0_415 | 49,848,112 | 94.64 | 2,418,012 | 4.59 | 20,613 | 0.039 | 47,774 | 0.091 | 336,069 | 0.638 |
| 168 | SPX0_464 | 47,014,069 | 94.51 | 2,324,249 | 4.67 | 17,692 | 0.036 | 43,233 | 0.087 | 344,972 | 0.693 |
| 169 | SPX0_482 | 50,193,003 | 96.08 | 1,676,587 | 3.21 | 18,565 | 0.036 | 35,149 | 0.067 | 316,301 | 0.605 |
| 170 | SPX1_036 | 51,909,660 | 98.01 | 669,000 | 1.26 | 16,076 | 0.030 | 52,294 | 0.099 | 314,728 | 0.594 |
| 171 | SPX0_290 | 58,812,121 | 82.17 | 12,091,036 | 16.89 | 50,522 | 0.071 | 63,199 | 0.088 | 554,384 | 0.775 |
| 172 | SPX0_205 | 56,479,537 | 95.65 | 2,167,726 | 3.67 | 17,627 | 0.030 | 37,406 | 0.063 | 347,192 | 0.588 |
| 173 | SPX0_217 | 56,887,893 | 95.10 | 2,510,336 | 4.20 | 19,115 | 0.032 | 43,002 | 0.072 | 361,489 | 0.604 |
| 174 | SPX1_222 | 57,271,003 | 96.16 | 1,874,975 | 3.15 | 18,147 | 0.030 | 42,136 | 0.071 | 352,176 | 0.591 |
| 175 | SPX1_302 | 58,009,791 | 95.58 | 2,251,341 | 3.71 | 19,224 | 0.032 | 42,224 | 0.070 | 369,199 | 0.608 |
| 176 | SPX2_215 | 55,165,118 | 96.42 | 1,671,347 | 2.92 | 17,809 | 0.031 | 39,255 | 0.069 | 321,734 | 0.562 |
| 177 | DPX0_013 | 54,103,313 | 97.37 | 1,115,210 | 2.01 | 18,452 | 0.033 | 28,782 | 0.052 | 297,399 | 0.535 |
